# A dormant TIL phenotype defines non-small cell lung carcinomas sensitive to immune checkpoint blockers

**DOI:** 10.1038/s41467-018-05032-8

**Published:** 2018-08-10

**Authors:** S. N. Gettinger, J. Choi, N. Mani, M. F. Sanmamed, I. Datar, Ryan Sowell, Victor Y. Du, E. Kaftan, S. Goldberg, W. Dong, D. Zelterman, K. Politi, P. Kavathas, S. Kaech, X. Yu, H. Zhao, J. Schlessinger, R. Lifton, D. L. Rimm, L. Chen, R. S. Herbst, K. A. Schalper

**Affiliations:** 1 Medical Oncology and Yale Cancer Center, New Haven, CT 06511 USA; 2 Department of Genetics, Yale School of Medicine, New Haven, CT 06511 USA; 3 Department of Pathology, Yale School of Medicine, New Haven, CT 06511 USA; 4 Translational Immuno-oncology Laboratory, Yale Cancer Center, New Haven, CT 06511 USA; 5 Immunobiology, Yale School of Medicine, New Haven, CT 06511 USA; 6 Yale School of Public Health, New Haven, CT 06511 USA; 7 Laboratory Medicine, Yale School of Medicine, New Haven, CT 06511 USA; 8 Department of Pharmacology, Yale School of Medicine, New Haven, CT 06511 USA

## Abstract

The biological determinants of sensitivity and resistance to immune checkpoint blockers are not completely understood. To elucidate the role of intratumoral T-cells and their association with the tumor genomic landscape, we perform paired whole exome DNA sequencing and multiplexed quantitative immunofluorescence (QIF) in pre-treatment samples from non-small cell lung carcinoma (NSCLC) patients treated with PD-1 axis blockers. QIF is used to simultaneously measure the level of CD3+ tumor infiltrating lymphocytes (TILs), in situ T-cell proliferation (Ki-67 in CD3) and effector capacity (Granzyme-B in CD3). Elevated mutational load, candidate class-I neoantigens or intratumoral CD3 signal are significantly associated with favorable response to therapy. Additionally, a “dormant” TIL signature is associated with survival benefit in patients treated with immune checkpoint blockers characterized by elevated TILs with low activation and proliferation. We further demonstrate that dormant TILs can be reinvigorated upon PD-1 blockade in a patient-derived xenograft model.

## Abstract (web summary)

Immune checkpoint blockade (ICB) only induces tumour response in a subset of non-small cell lung carcinomas (NSCLC). Here the authors do whole genome sequencing and multiplexed quantitative immunofluorescence on patient samples and identify a “dormant” T-cell signature associated with sensitivity to ICB.

## Introduction

Immunomodulatory therapies using monoclonal antibodies to block the co-inhibitory receptors programmed death-1 (PD-1) and cytotoxic T-lymphocyte associated protein 4 (CTLA-4) have revolutionized the treatment of diverse tumor types, including non-small cell lung cancer (NSCLC). Treatment with PD-1 axis blockers induces tumor response in approximately 20% of unselected patients with advanced NSCLC^1–4^. The combination of PD-1 and CTLA-4 blockers results in greater anti-tumor effect than monotherapy regimens in melanoma, and is currently being evaluated in NSCLC^5–8^. Despite unprecedented durability of response, the majority of NSCLC patients receiving PD-1 axis blockers do not derive clinical benefit. Clearly, predictive biomarkers to select patients for these therapies are required. In addition, understanding the biological determinants that mediate sensitivity and resistance to immune checkpoint blockade could support design of optimal treatment modalities. Diverse studies have shown that tumor PD-L1 protein expression using chromogenic immunohistochemistry (IHC) can enrich for responders to PD-1 blocking agents^1–4^. Expression of PD-L1 in NSCLC (and other tumor types) is associated with increased tumor immune infiltration and local IFN-γ production, suggesting its adaptive modulation in the tumor microenvironment^9,10^. Although four PD-L1 IHC tests have been approved by the US Food and Drug Administration for clinical use (e.g., 22C3, 28-8, SP263, and SP142), there can be discordance between results from different assays, and a negative test does not preclude response to PD-1 axis inhibitors. Additional factors have also been associated with response to PD-1 axis blockade including increased CD8+ tumor infiltrating lymphocytes (TILs)^11,12^, TIL PD-1 expression^11^, clonally expanded T-cell populations^11^ and elevated somatic mutations or candidate MHC class-I neoantigens^12–14^. The biological link between these factors and potential predictive value of combining them remain uncertain.

Recent studies have shown that an elevated tumor mutational load or predicted class-I neoantigen content is associated with higher response rate and survival to PD-1 or CTLA-4 blockade in melanoma^14–17^. Similar findings have been reported in patients with mismatch-repair deficient carcinomas and NSCLCs treated with PD-1 axis blockers^12,13^. This supports the hypothesis that tumors with more mutations likely generate more neoepitopes, which can be recognized by TILs. Treatment with immune checkpoint blocking antibodies can stimulate neoantigen-specific TILs and mediate tumor regression. Additional studies indicate that neoantigens present at higher allelic frequency within the tumor population (e.g., “clonal” neoantigens) are biologically more relevant^18^. However, neoantigen specific lymphocytes have been found at relatively low levels and only against a few of the mutant epitopes detected in the tumor^13,17,19–21^. In addition, there are tumors with relatively low mutational burden which are sensitive to immune checkpoint blockers such as renal cell carcinomas^22^. Previous reports from melanoma, NSCLC, and mismatch-repair deficient carcinomas also indicate that some tumors harboring extremely elevated mutational load do not derive clear benefit from PD-1 and CTLA-4 blockade^12,13,16^. Analyses of the The Cancer Genome Atlas (TCGA) dataset has linked the presence of elevated mutations or candidate MHC class-I neoantigens with increased levels of perforin and granzyme-A mRNA transcripts, suggesting a link between the level of genomic alterations and effective anti-tumor immune responses^23^. However, the cell types producing these cytolytic enzymes were not determined and the association was evident only in some tumor types such as cervical (HPV-positive) carcinoma, lung, and colorectal adenocarcinomas; but not in melanoma, bladder and lung squamous cell carcinomas. Additional studies using the TCGA database showed that lung squamous tumors display reduced markers of effective immune surveillance compared to lung adenocarcinomas despite having comparable candidate neoantigen levels^18^. The lower anti-tumor immune response in squamous carcinomas was associated with low expression of antigen presentation genes, suggesting that mechanisms different from the mutational load can modulate the anti-tumor immune response in this malignancy. Using whole exome DNA sequencing and multiplexed quantitative in situ immunofluorescence, we studied the association between the mutational landscape, local anti-tumor T-cell responses and clinical benefit to immune checkpoint blockers in patients with NSCLC. We identified and experimentally validated a “dormant” TIL signature associated with sensitivity to PD-1 axis blockade that is independent from the tumor mutational burden and PD-L1 expression.

## Results

### Mutations neoantigens and immune checkpoint blockade

**Fig. 1:**
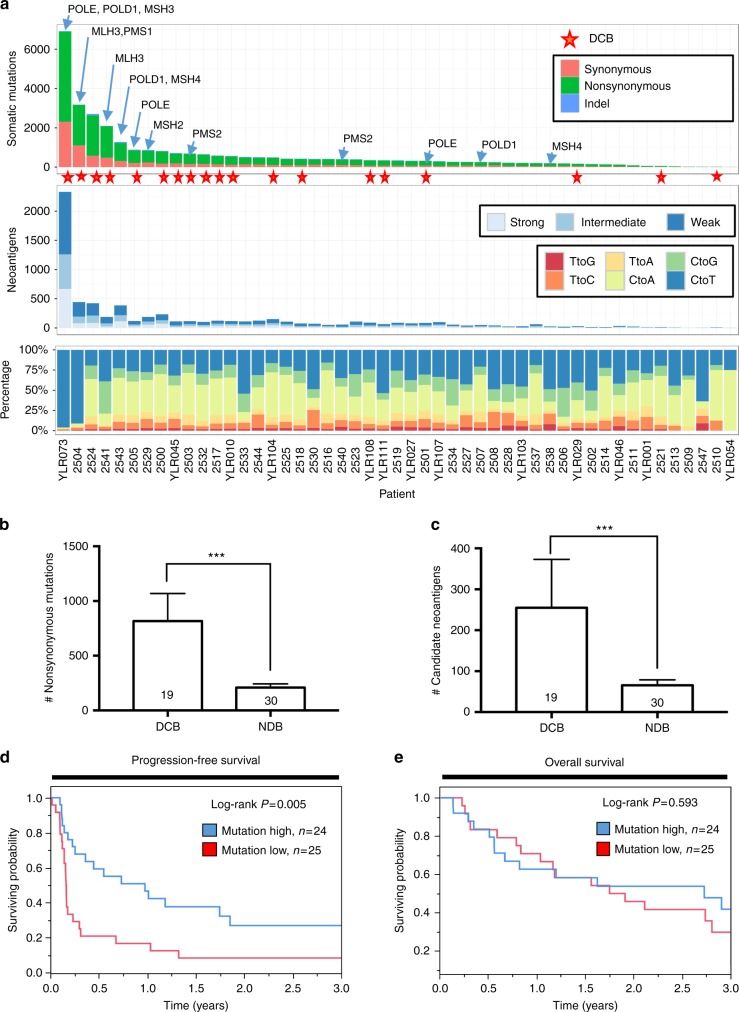
Association between mutations, candidate class-I neoantigens and benefit from immune checkpoint blockers in NSCLC. **a** Chart showing the number of somatic mutations (upper panel), candidate MHC class-I neoantigens (middle panel) and frequency of specific nucleotide substitution (lower panel); and association with DCB to immune checkpoint blockade (red star) in 49 NSCLC cases. The presence of mutations in genes associated with DNA repair is indicated with arrows over each case. The specific variant type is indicated within the chart. **b**, **c** Association between the mutational load (**b**) and predicted MHC class-I neoantigens. Error bars indicate S.E.M. ***P < 0.001, Mann–Whitney test. **c** With durable clinical benefit (DCB) and no durable benefit (NDB) to immune checkpoint blockade. The number of cases in each group is indicated in each bar. **d**, **e** Association between the mutational load and 3-year progression-free survival (**d**) and overall survival (**e**) after treatment with immune checkpoint blockers. The median mutational number was used as stratification cut point. The number of cases in each group and log-rank *P* value are indicated within each chart

To characterize the mutational landscape of our cohort and its association with clinical response, we performed whole exome DNA sequencing analysis of pre-treatment formalin-fixed paraffin-embedded (FFPE) NSCLC samples from 49 patients treated with immune checkpoint blockers. The mean target coverage was 206.3× and 97.08% of nucleotides read at least 20×. The average somatic mutation load was 633.27 (range 10–6926) with a median of 346. The average nonsynonymous mutational load was 444.7 (range 5–4577) with a median of 252. The association between the total mutational load and the number of nonsynonymous variants was high (Spearman’s correlation coefficient [*R*] = 0.99, *P* < 0.0001). As shown in Fig. 1a, cases with a mutational burden above the median of the cohort showed a 3.6-fold higher frequency of durable clinical benefit (DCB) than cases with lower mutational content (60 vs 16.7% with DCB, respectively, *P* = 0.05, Fisher’s extact test). In addition, highly mutated tumors had 2.6-fold higher frequency of variants in genes associated with DNA repair (e.g., MLH3, MSH6, POLD1, POLE, etc.) than cases with low mutations, but this difference did not reach statistical significance (32% vs 12.5%, respectively, *P* = 0.31, Fisher’s extact test). As expected for a NSCLC population, the majority of cases displayed a mutational signature with predominance of C>A transversions, previously reported to be associated with tobacco exposure (Fig. 1a)^13,24^.

We then identified candidate class-I neoantigens through a bioinformatic pipeline including: (i) In silico translation of the nonsynonymous mutant sequences into 17-mer polypeptides flanking the mutant amino acid; (ii) calculation of mutant nonamers with IC_50_ below or equal to 500 nM to bind patient-specific class-I HLA alleles; and (iii) determination of the predicted recognition of mutant sequences by T-cells^13,23,25^. As shown in Fig. 1b and Supplementary Table 1, we identified a mean of 138.8 candidate MHC class-I neoantigens per case (range 2–2331), 25.9% of which were predicted to be high affinity binders (e.g., with a calculated IC_50_ ≦ 50 nM), 26.8% intermediate binders (with a calculated IC_50_ 50–150 nM) and 47.3% weak binders (with a calculated IC_50_150–500 nM). There was a high correlation between the mutational load and predicted class-I neoantigen content (Spearman’s *R* = 0.95, *P* < 0.0001), as reported^12,23^. Both the nonsynonymous mutational load and the number of in silico predicted MHC class-I neoantigens were significantly higher in cases with DCB than in patients without DCB after immune checkpoint blockade (*P* = 0.0004 and *P* = 0.0009, respectively, Mann–Whitney test [Fig. 1b, c]). However, some patients with low mutations/neoantigens achieved DCB. A comparable result was obtained using the total mutational burden (Supplementary Figure 1). Using the median value as stratification cut-point, the sensitivity of the nonsynonymous mutational load to predict DCB was 78.9% and the specificity was 66.6%. The most frequently mutated cancer-related gene in the cohort was TP53 (57.1%). We found no significant difference in the relative frequency of specific oncogenic mutations (Supplementary Figure 2), global loss of heterozygosity (LOH) (Supplementary Figure 3) or in the amount of copy number variations (CNVs, Supplementary Figure 4) between patients with and without DCB. Increased nonsynonymous mutational load was significantly associated with longer progression free survival (Fig. 1d, *P* = 0.005, test). However, we found no significant association between the level of mutations and overall survival at 3-years using the same stratification cut-point (Fig. 1e, log-rank *P* = 0.59, log-rank test). An equivalent association with survival was seen using the number of candidate class-I neoantigens (Supplementary Figure 5).

### HLA binding and T-cell responses to mutant neopeptides

**Fig. 2:**
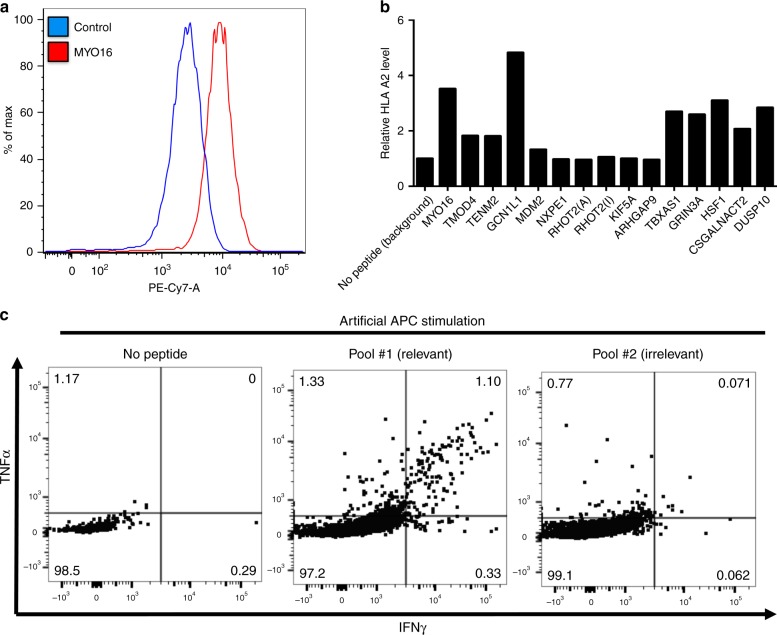
Experimental validation of predicted MHC class-I neoantigens detected in NSCLC. **a** Representative flow cytometry histogram showing the relative PE-Cy7 fluorescence of surface HLA-A2 in LCL-174 cells in the control condition (blue) or after incubation with recombinant MYO16 mutant neopeptide (red). (**b**) Distribution of stabilization signal scores for each recombinant neopeptide binding to HLA-A2 protein in LCL-174 cells. Scores are expressed a fold change relative to the signal obtained using the negative control. The data presented correspond to neoepitopes identified in three different tumor samples from one patient (primary tumor, metastasis, and recurrence). Each score obtained was averaged and a ratio was calculated respect to the average of the negative control peptide signal. Data in (**a**, **b**) is representative from two individual experiments. **c** FACS plot showing the levels of intracellular IFN-γ and TNF-α measured by flow cytometry in cultured CD8+ T-cells obtained from peripheral blood of the NSCLC patient after stimulation with artificial APCs and no peptide (left panel), a pool containing eight recombinant mutant neopeptides preincubated with mature autologous APCs (pool #1 relevant, center panel) and another pool of six mutant neopeptides not preincubated with autologous APCs (pool #2 irrelevant, right panel). Data in (**c**) is representative from two replicate experiments

Experimental validation of the HLA binding capacity of in silico predicted class-I mutant neoantigenic peptides found in NSCLC (Table 1) was performed by measuring the stabilization of HLA-A2 protein after incubation of B lymphoblastoid LCL-174 cells lacking MHC-II genes and TAP proteins, with recombinant mutant 9-mer peptides. As shown in Fig. 2a, b, 9 out of 13 (67.9%) predicted mutant neopeptides found in tumor samples obtained over the course of treatment in a patient with HLA-A*0201 HLA type showed positive surface fluorescence signal, indicating effective peptide-class-I MHC binding. The predicted affinity of the mutant peptide-HLA-A2 interaction was correlated with the in vitro stabilization scores, showing the highest HLA-A2 signal in those cases with the lowest predicted IC_50_. Notably, four out of five mutant peptides with predicted IC_50_ > 100 nM failed to show detectable HLA stabilization, indicating limited actual binding. In addition, the predicted binding affinity of the mutant relative to the non-mutated peptides was prominently higher (mean IC_50_ 103.6 ± 33.2 for the mutant peptides vs 2541 ± 1218 for the wild type sequences).

**Table 1:**
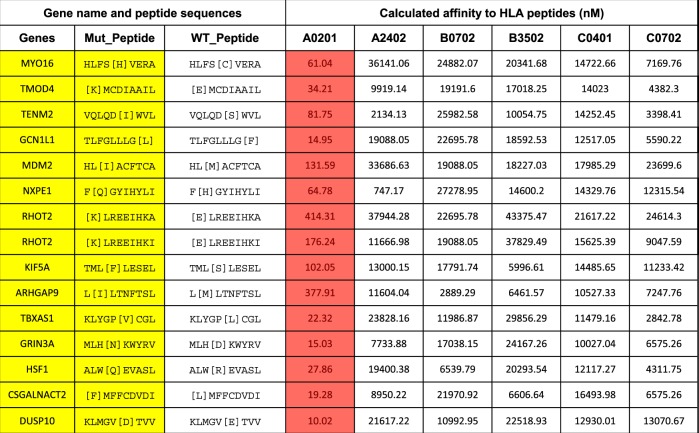
Table showing the gene name, mutant neopeptide (yellow), wild type peptide sequence (yellow) and predicted nanomolar affinity for the HLA-A/B/C found in NSCLC samples from a patient with HLA*A2 type over the course of disease. The calculated affinity of the mutant peptides for HLA*A2 is highlighted with red colored cells

We then tested whether the predicted mutant neopeptides could elicit a functional T-cell response in lymphocytes derived from peripheral blood from the same NSCLC patient. To this end, we cultured peripheral blood mononuclear cells (PBMCs) and sepatared monocytes for in vitro maturation (see 'Methods' section). Neoantigen-specific T-cells were expanded by stimulation with a pool of eight tumor neopeptides (peptide pool #1) exhibiting a range of predicted HLA-A2 binding affinities in the presence of autologous mature dendritic cells. The cell preparations were subsequently stimulated with artificial antigen presenting cells (APCs) K562 expressing HLA-A2 and 4-1BBL in the presence of peptide pool #1 followed by re-stimulation after one week with the same APCs in the presence or absence of the peptide pool. Additionally, a second peptide pool (peptide pool #2) containing six candidate neoepitopes that were not used in the initial T-cell expansion was used as an irrelevant peptide set for negative reactivity control. Intracellular cytokine flow cytometry was used to measure IFN-γ and TNF-α production in CD8+ T-cell subpopulations. As shown in Fig. 2c, re-stimulation with the peptide pool #1 induced a positive response of T-cells dually expressing IFN-γ and TNF-α in 1.1% of the CD8+ population, indicative of T-cell recognition of the mutant neopeptides. This change was >15-fold higher than in T-cells stimulated with APCs in the absence of peptides or in the presence of the irrelevant peptide pool #2. In a separate experiment using individual peptides, five of the eight peptides in pool #1 elicited a detectable CD8+ T-cell response.

### Mutational load oncogenic drivers and tobacco consumption

**Fig. 3:**
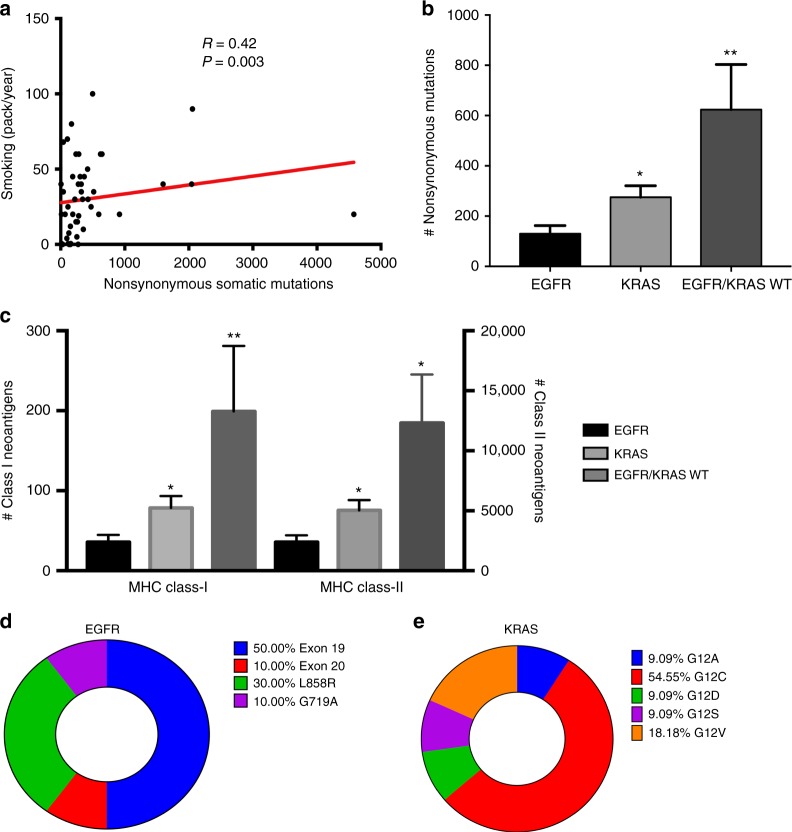
Association between the mutations, predicted MHC class-I neoantigens, major oncogenic drivers and tobacco consumption. **a** Association between the mutational load and the level of cigarette smoking in lung cancer patients treated with immune checkpoint blockers. *R* = Spearman’s rho rank correlation coefficient. **b**, **c** Charts showing the number of nonsynonymous mutations (**b**) or candidate HLA class-I and class-II neoantigens. **c** In lung tumors treated with immune checkpoint blockers harboring mutations in EGFR (*N* = 8), KRAS (*N* = 11) or lacking mutations in both oncogenes (*N* = 30). Error bars indicate S.E.M. *P < 0.05; **P < 0.01, Mann–Whitney test. **d**, **e** Frequency of the specific variants identified in NSCLCs with EGFR (**d**) and KRAS mutations (**e**) *Mann–Whitney *P* value < 0.05; **Mann–Whitney *P* value < 0.01

As expected, the mutational load was positively correlated with the amount of cigarrete consumption (Spearman’s *R* = 0.42, *P* = 0.01, Fig. 3a). Tumors harboring activating mutations in EGFR had significantly lower somatic mutations and predicted MHC class-I and class-II neoantigens than KRAS-mutant and EGFR/KRAS wild type tumors (*P* < 0.01, Mann–Whitney test Fig. 3b, c). Although the tumors lacking mutations in EGFR and KRAS showed a higher mutational/candidate neoantigen level than KRAS-mutant carcinomas, this difference was not statistically significant. As expected, Exon 19 deletions were the most common EGFR mutations (50% of EGFR mutant cases) and all KRAS variants were located in codon 12 with G12C being the most frequently detected (54% of KRAS mutant tumors) (Fig. 3d, e).

### TILs and benefit from immune checkpoint blockers

**Fig. 4:**
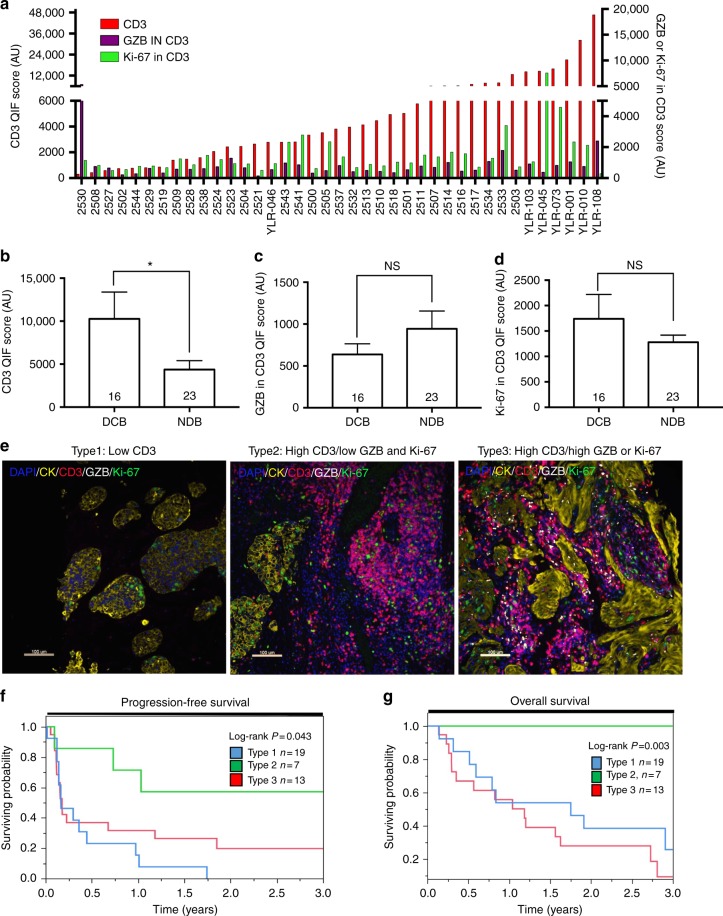
Association between local T-cell infiltration and activation/proliferation and benefit from immune checkpoint blockers in NSCLC. **a** Distribution of in situ CD3 (red, left Y axis), T-cell GZB (magenta, right Y axis) and T-cell Ki-67 signal (green, right Y axis) in lung tumors from patients treated with immune checkpoint blockers. **b**–**d** Association between the level of CD3 (**b**), T-cell GZB (**c**), and T-cell Ki-67 (**d**) with durable clinical benefit (DCB) or no durable benefit (NDB) to immune checkpoint blockade. The number of cases in each group is indicated within each bar. NS = not significant with Mann-Whitney *P* > 0.05. *Mann–Whitney *P* value . Error bars indicate S.E.M. **e** Representative multiplexed fluorescence pictures showing lung tumors with a type 1 TIL pattern containing low CD3 level (left panel), a type 2 pattern with high CD3 but low T-cell GZB/Ki-67 (center panel); and a type 3 TIL phenotype with high CD3 and elevated T-cell GZB/Ki-67 (right panel). The color assigned to each marker is indicated within each caption. Bar = 100 μm.**f**, **g** Kaplan–Meier graphical analysis of 3-year progression free survival (**f**) and overall survival (**g**) of lung cancer cases treated with immune checkpoint blockers according to their TIL phenotype panel. The number of cases in each group and the log-rank *P* value is indicated in the chart

We measured the levels of T-cells and in situ T-cell activation/proliferation using multiplex quantitative immunofluorescence (QIF) in 39 cases from the cohort with available tumor tissue. Our QIF panel included DAPI to highlight all cells/nuclei in the sample, cytokeratin (CK) to stain tumor epithelial cells, CD3 for T-lymphocytes, granzyme-B (GZB) for T-cell activation and Ki-67 for cell proliferation. We then measured the level of CD3 as a metric of T-cell infiltration and the amount of GZB and Ki-67 in CD3-positive cells as indicators for T-cell activation and proliferation, respectively. The design and performance of this panel was validated using control FFPE preparations of human tonsil, lymph node and unstimulated (control) human PBMCs or PBMCs stimulated for 72 h with anti-human CD3 monoclonal antibodies (clone OKT-3) (Supplementary Figure 6). The level of CD3 signal showed a continuous distribution and a wide range going from virtually no TILs to prominent T-lymphocyte infiltration (Fig. 4a). As shown in the Supplementary Figure 7, the stromal CD3 signal (CD3 in CK-negative areas) was 2.5 fold higher (*P* < 0.001, Mann–Whitney test) than in the tumor (CD3 in CK-positive areas), indicating that the majority of the T-cells were located in the stromal compartment. However, the stromal and tumor CD3 levels were positively correlated. The level of CD3 signal was not correlated with the level of T-cell GZB (Spearman’s *R* = 0.23, *P* = 0.14) and only modestly correlated with T-cell proliferation (Spearman’s *R* = 0.41, *P* = 0.01). The level of T-cell infiltration was 2.4 fold higher in cases with DCB (*P* = 0.02; Mann-Whitney test Fig. 4b). The level of TIL activation or proliferation was not associated with the presence or absence of DCB **(**Fig. 4c, d**)**.

Stratification of the cases into three groups based on their median CD3, T-cell GZB, and T-cell Ki-67 levels identified tumors with a “dormant” TIL phenotype (or Type 2) characterized by elevated CD3 but low T-cell GZB and low T-cell Ki-67 as the one with the highest rate of DCB (86% of cases with DCB) and longest progression free and overall survival (Fig. 4e–g). Representative multicolor fluorescence pictures of the tumors showing the three distinct QIF-based TIL patterns are shown in Fig. 4e.

**Fig. 5:**
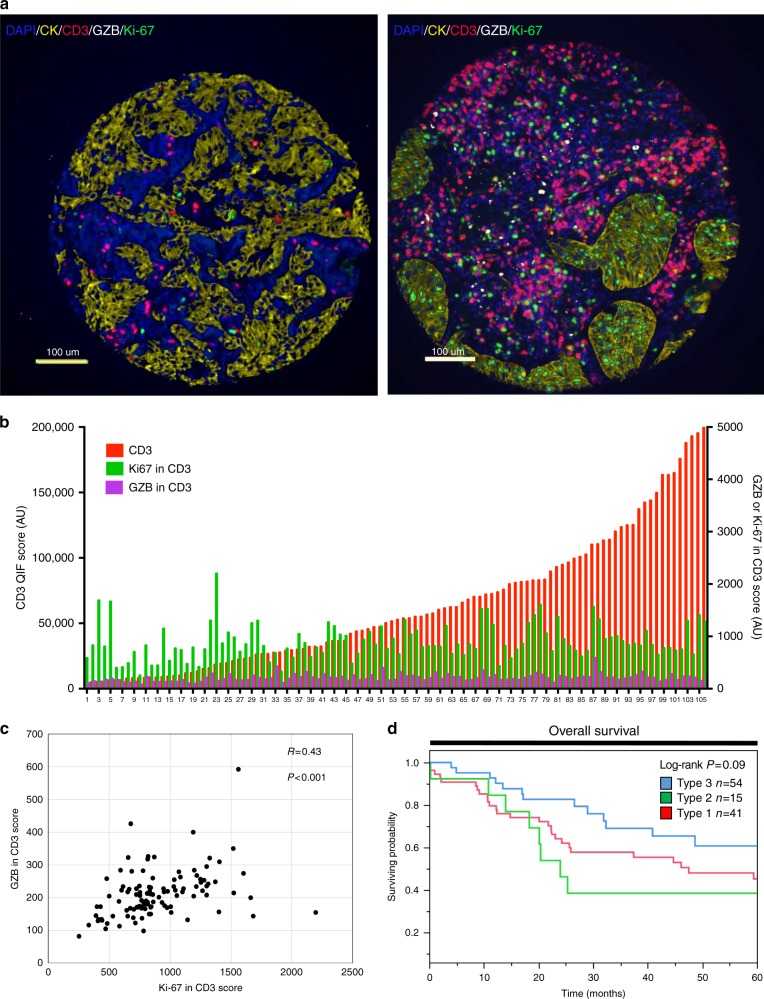
Association between local T-cell infiltration and activation/proliferation and survival in NSCLC patients not treated with immune checkpoint blockade. **a** Immunofluorescent staining of a lung tumor with low (**a**) and high (**b**) T-cell activation/proliferation. Slides were simultaneously stained with a multiplex QIF panel containing CD3 (red), Ki-67 (green), GZB (white), DAPI (blue), and cytokeratin (yellow). Bar = 100 μm. **b** Distribution of in situ CD3 (red, left Y axis), T-cell GZB (magenta, right Y axis) and T-cell Ki-67 signal (green, right Y axis) in lung tumors from patients not receiving immune checkpoint blockers. **c** Association between the level of T-cell GZB and T-cell Ki-67 in the cohort. *R* = Spearman’s correlation coefficient. The *P* value for the correlation is indicated within each chart. **d** Kaplan–Meier graphical analysis of 5-year overall survival of NSCLC cases not treated with immune checkpoint blockers according to their TIL activation subtypes. A type 1 TIL pattern was with low CD3, a type 2 pattern with high CD3 but low T-cell GZB/Ki-6; and a type 3 TIL phenotype with high CD3 and elevated T-cell GZB/Ki-67. The number of cases in each group and the log-rank *P* value is indicated in the chart

To assess the specificity of the association between the QIF-based TIL signatures and treatment with immune checkpoint blockers, we measured the levels of T-cell infiltration, activation, and proliferation in a retrospective collection of 110 stage I–IV NSCLCs not treated with immunotherapy and represented in tissue microarray (TMA) format (Fig. 5). Similar to the treated cases, the level of CD3+ cells in lung tumors was variable and not directly associated with the markers of T-cell activity/proliferation (Fig. 5b). There was a moderate positive association between the levels of T-cell GZB and Ki-67 (Spearman’s *R* = 0.43, Fig. 5c). However, in this population the group with the “dormant” TIL phenotype (Type 2) was not associated with survival benefit, supporting the notion that the presence of inactive TILs is associated with better outcome only in patients treated with immune checkpoint blockers. Notably, the group with a Type 3 TIL profile displaying elevated CD3+ TILs and high lymphocyte GZB and/or Ki-67 showed a clear trend toward better survival in this population (*P* = 0.09, log-rank test, Fig. 5d).

### PD-1 blocking antibodies can reinvigorate dormant TILs

**Fig. 6:**
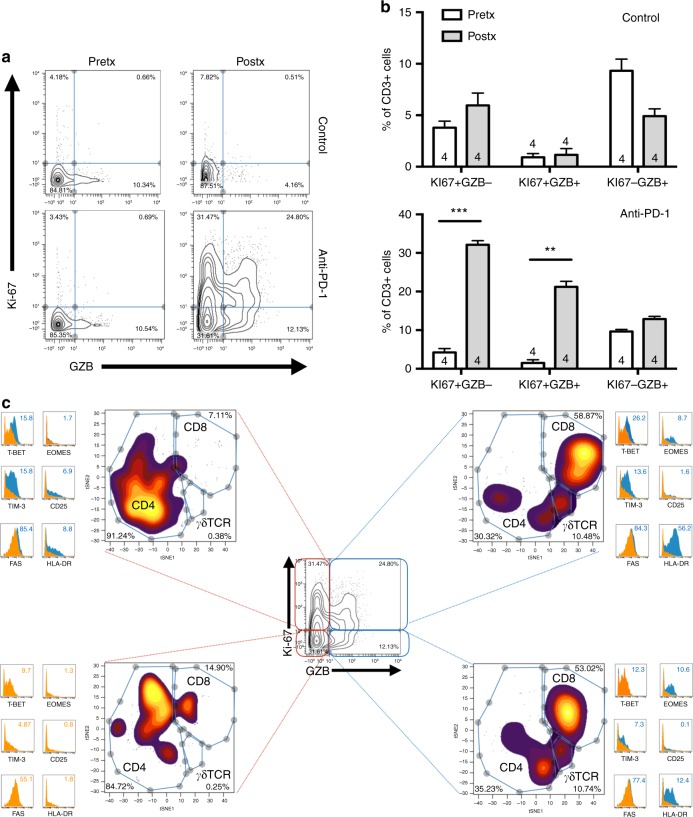
T-cell reinvigoration with PD-1 blocking antibodies. Surgically resected primary NSCLCs were engrafted subcutaneously in the flank of NOD-scid IL2rgc-/- mice. Mice were treated intraperitoneally with anti-hPD-1 mAbs (anti-PD-1) or PBS (Control) at days 5 and 10. At day 12 mice were sacrificed and tumor tissues were mechanically digested and single cells studied by mass cytometry. **a**, **b** Representative graphs depicting the levels of Ki-67 and GZB expression in TILs (CD3+) before engraftment (Pretx) and at the moment of sacrifice (Postx) in control and anti-PD-1 treated tumor-bearing mice. Dot plot a is representative of four experiments shown in (**b**). Error bars indicate S.E.M. **P < 0.01; ***P < 0.001, Mann–Whitney test. The number of independent experiments is indicated within the bars. **c** viSNE map of each biaxial plot of Ki-67 vs GZB quadrant. Three subpopulations were clustered using viSNE: CD4, CD8, and γδTCR T cells. Expression profile of each quadrant is depicted in small panels. Numbers indicate the median mass intensity for each marker. Expression of quadrant Ki-67-GZB- was used as a reference

To experimentally demonstrate the cytolytic activation/proliferation of “dormant” TILs upon PD-1 axis blockade, we engrafted surgically resected primary lung cancer explants subcutaneously in immune deficient mice and administered intraperitoneally anti-PD-1 monoclonal antibodies. Because we did not perform any tumor passage in mice, this surgical lung cancer explants contain tumor and also original patient-derived TILs. After treatment, cells were isolated from the resected tumor specimens and analyzed using mass cytometry. As shown in Fig. 6a, b, human CD3+ TILs displaying low basal GZB and low Ki-67 prominently increased the levels of both markers in animals treated with PD-1 blockade but not in control mice. The largest difference before and after treatment was seen in T-cells expressing Ki-67 alone or Ki-67 plus GZB (Fig. 6b). Notably, Ki-67 was predominantly increased in CD4+ T-lymphocytes and GZB was higher in the CD8+ population (Fig. 6c). Further characterization of TILs after PD-1 blockade showed elevated levels of T-BET and TIM-3 in T-cells with increased proliferation and higher levels of EOMES and HLA-DR in the GZB-high subpopulation. The fraction of T-cells showing increased Ki-67 and GZB was predominantly CD8+ with relatively high levels of T-BET, EOMES, TIM-3, and HLA-DR.

### Mutations PD-L1 expression and T-cells in NSCLC

**Fig. 7:**
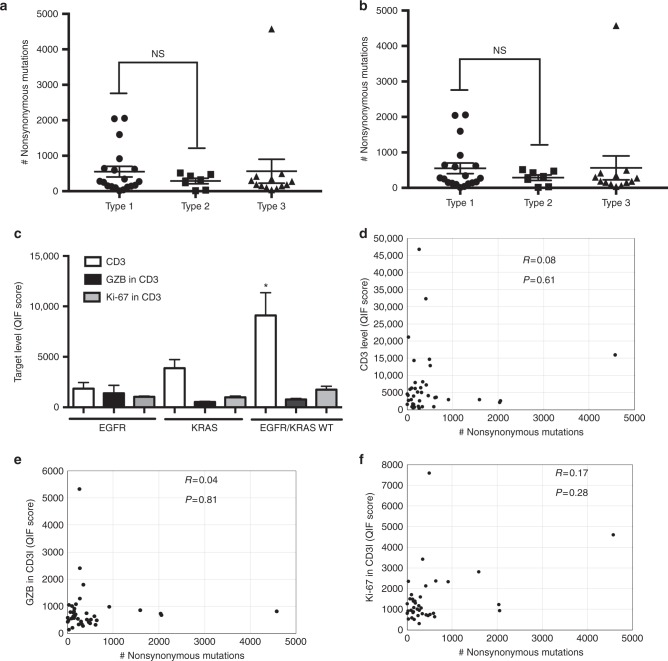
Association between mutations and tumor immune infiltration and proliferation and cytolytic activity in NSCLC. **a**, **b** Association between the mutational load (**a**) or predicted class-I neoantigens (**b**) and specific TIL patterns found in lung tumors from patients treated with immune checkpoint blockers. NS = not significant with Mann–Whitney *P* > 0.05. **c** Chart showing the level of CD3 (white bars), T-cell GZB (black bars), and T-cell Ki-67 (gray bars) in lung tumors from patients treated with immune checkpoint blockers harboring mutations in EGFR (*N* = 8), KRAS (*N* = 11) or lacking mutations in both oncogenes (*N* = 30). Error bars indicate S.E.M. *P < 0.05; NS = not significant (*P* > 0.05), Mann–Whitney test. **d**, **f** Association between the mutational load and the level of CD3 (**d**), T-cell GZB (**e**), and T-cell Ki-67 (**f**) in lung cancer patients treated with immune checkpoint blockers. *R* = Spearman’s correlation coefficient. The *P* value for the correlation is indicated within each chart

Notably, the level of somatic mutations and predicted class-I neoantigens were not significantly different across the three TIL NSCLC subtypes and the majority of cases with very high mutational load or candidate neoantigens displayed a type 1 pattern (Fig. 7a, b). EGFR mutant cases showed significantly lower levels of T-cell infiltration than tumors lacking EGFR and KRAS mutations (*P* = 0.03, Mann–Whitney test Fig. 7c). KRAS mutant tumors showed approximately 2-fold higher CD3 signal than those with EGFR mutations, but this difference was not statistically significant. The level of GZB and Ki-67 in T-cells was not different across major molecular variants of lung adenocarcinoma. There was a low association between the nonsynonymous mutational load and the level of T-cell infiltration, T-cell activation and proliferation (Spearman’s *R* = 0.04–0.17, Fig. 7d–f). Consistent with this, the effect of the mutational load and the TIL groups were independently associated with overall survival in a multivariable Cox proportional hazard model (Supplementary Table 5). As shown in the Supplementary Figure 8, the mutational burden (Supplementary Figure 8A) and the TIL subtypes (Supplementary Figure 8B) were not associated with the levels of tumor PD-L1 protein expression as measured using chromogenic IHC.

To further explore the association between the mutational load and tumor inflammation, we analyzed the NSCLC cases from the TCGA collection. As shown in Supplementary Figure 9, the mutational load was only marginally correlated with the level of adaptive immunity transcripts in lung adenocarcinomas including CD8A, CXCL10, GZMB, IFNG, and STAT1 (Spearman’s *R* = 0.14–0.3) and there was no relationship with other TIL markers such as CD3, CD4, PRF1 and MS4A1 (Spearman’s *R* = −0.08–0.006, Supplementary Figure 9A). Consistent with previous observations^18,23^, there was no association between the mutational load and the adaptive immunity markers in lung squamous cell carcinomas (Spearman’s *R* = −0.03–0.18, Supplementary Figure 9B).

## Discussion

Using whole exome DNA sequencing and multiplexed quantitative TIL immunoprofiling we show that the tumor mutational load and candidate class-I neoantigens are significantly associated with durable responses to immune checkpoint blockers, but not with T-cell tumor infiltration, TIL function and overall survival in NSCLC. In addition, we identified a TIL signature in pre-treatment tumor samples characterized by a dormant phenotype (i.e., high CD3+/low GZB/low Ki-67) that is associated with survival benefit to immune checkpoint blockers independent from the tumor mutational load and PD-L1 expression.

Our results are consistent with previous studies showing a significant association between the mutational load or predicted MHC class-I neoantigens and response to immune checkpoint blockers^13–17^. However, our data indicate that this association is not very strong (78.9% sensitivity and 66.6% specificity) and may not translate into improved overall survival. The biological determinants of this apparent contradiction are uncertain, but could be related to the observation that patients with elevated tumor mutations tend to be smokers who more commonly display poorer health with prominent co-morbidity. In addition, genomically unstable lung tumors may adapt faster to immune pressure imposed by checkpoint blockade than tumors with low mutational burden. In support of this notion, we found no significant association between the mutational load and the level of tumor T-cell infiltration or lymphocyte activation/proliferation suggesting that advanced mutation/neoantigen-rich lung carcinomas are equipped with potent immune suppressive mechanisms. Alternatively, we should consider the possibility that mutant neopeptides are only one component of the immunogenic drivers in lung cancer and other mechanisms could also be involved in the anti-tumor effects of immune checkpoint blockers. For instance, aberrant expression of proteins or peptides with specific post-translational modifications detected in cancer (e.g., phosphorylation, acetylation, and citrullination) have been shown to trigger CD4 and CD8 T-cell responses^26–30^. Supporting this possibility, the estimated mutational/neoantigen load was not clearly associated with anti-tumor immunity in bladder urothelial carcinomas treated with the anti PD-L1 drug atezolizumab^31^ and with response to PD-1 blockade in melanoma^14^. Additional mechanisms for tumor immune recognition may also have a role in these effects, including cytosolic responses to DNA damage, altered/foreign nucleic acids or cell stress^32,33^. Distinct genomic mutational patterns (e.g., tobacco mutagen signature), microbial/viral exposures, and unique immune features of lung cancer could explain the differences with a previous study in colorectal carcinomas showing association between the mutational load, predicted neoantigens and local lymphocyte responses^34^. However, and consistent with our results, this study found that the neoantigen load was associated with CD45RO+ cells, but not with CD3+ T-cells and CD8+ cytotoxic cell density^34^. Larger studies carefully studying the genomic landscape and immune contexture in lung cancer using localized protein measurements will be required to clarify this point.

Recent publications have suggested the use of the tumor mutational load and candidate neoantigen content as predictive biomarkers for immune checkpoint blockers and for identification of personalized targets for development of peptide/nucleic acid vaccines and adoptive cell therapies^15,35,36^. Although diverse studies interrogating class-I neoantigens in human malignancies have used similar methods (e.g., whole exome DNA sequencing and netMHC algorithm), there is currently no standard or widely accepted approach. Refinement of relevant neoantigen identification incorporating the mutant allelic frequency/clonality^18^, expression levels of the mutant transcripts^37^ and functional assays to detect antigen specific cells, T-cell peptide recognition or binding of the mutant peptides to HLA-proteins have been proposed^12,18–21,38^^.^ Unfortunately, these approaches require the use of sophisticated technical/bioinformatic resources and studies directly comparing their performance using clinical endpoints are lacking. In addition, most available strategies to predict and/or validate neoantigens rely on the particular patient HLA-types.

Recent studies looking for neoantigen specific T-cells in cancer patients using tetramer technologies or in vitro stimulation of T-cells with mutant sequences have shown that neoantigen-specific T-cell reactivity is generally low and limited to just a few of the mutant epitopes present in each tumor^13,19–21^. In melanoma patients, naive peripheral blood T-cells obtained from healthy donors were able to recognize mutant neoantigens in HLA-matched patients with melanoma that were neglected by the autologous patient T-cells^38^. This indicates a complex regulation of antigen-specific cells in cancer patients and reveal possible limitations of using the patient T-cell reactivity as endpoint for demonstrating the immunogenic capacity of predicted neoepitopes.

In our study, we used a pipeline to identify candidate class-I neoantigens based on their predicted affinity with patient-matched HLA-proteins and recognition by the T-cell receptor. In addition, we validated the strategy using an in vitro assay to detect HLA membrane stabilization upon challenging immune-selected lymphoblastoma cells with the recombinant mutant peptides. We also demonstrated T-cell reactivity against the mutant neoepitopes identified in a NSCLC using cultured lymphocytes extracted from peripheral blood of the same patient. Our preliminary results indicate that nearly 2/3 of candidate neoantigens predicted in the background of a patient with HLA*A2 type can effectively bind the HLA-proteins. However, the establishment of assays and evaluation of performance of predicted neopeptides in cases with other HLA-alleles that are less common or not well studied is uncertain and will require further development.

The nonsynonymous mutational load and predicted class-I neoantigen content in our study were similar to a previous report including 34 advanced NSCLC cases treated with PD-1 blocking antibodies (median of 256 vs 200 nonsynonymous mutations per genome; and 72 vs 112 candidate class-I neoantigens per tumor^13^). Our results are also similar to other studies using TCGA sequencing data from NSCLC^23,24^. Minor differences with previous reports are likely due to dissimilar approaches used for the variant calling and filtering. Our study also included 13 cases without available paired germline DNA in which the mutation load was established using tumor exome sequencing and reference libraries. Notably, the mutation load and candidate MHC class-I neoantigen content were comparable between cases with and without paired germline DNA and the association between mutations, neoantigens and response/survival was comparable when unpaired cases were excluded from the analysis (Figure 10). The latter confirms the robustness of the mutation analysis and indicates that the presence of cases with unpaired DNA did not impact the results.

Diverse metrics of tumor immune infiltration including the amount of CD8+ cytotoxic T-cells or RNA signatures of T-effector cells have been linked with clinical benefit to CTLA-4 and PD-1 axis blockers^1,11,12,17,31^. Our results using spatially resolved QIF measurements expand these observations and demonstrate a prominent role of pre-existing CD3+ TILs in the anti-tumor effect induced by immune checkpoint blockers in lung cancer. Additional studies are ongoing to clearly define the subpopulation of lymphocytes mediating these responses (e.g., specific CD4+ vs CD8+ subpopulations, expression of additional targets).

We studied two cohorts of patients with NSCLC and found limited correlation between the T-cell signal and the level of T-cell cytolytic potential/proliferation, confirming independence of the markers. Notably, a “dormant” T-cell phenotype characterized by elevated T-cells with low activation and proliferation markers in the tumor bed was significantly associated with longer survival only in the cohort of patients treated with immune checkpoint blockers. This finding is consistent with a recent report in melanoma showing that pre-treatment tumor samples with an exhausted CD8+ TIL phenotype characterized by high PD-1/CTLA-4 and low IL-2 and TNF-α was predictive of response to PD-1 blockade^39^. However, measurement of this T-cell signature using multi-color flow cytometry requires extraction of lymphocytes from fresh tumor tissue and in vitro stimulation with PMA and ionomycin. Our TIL signature was validated and standardized for use in conventional biopsy (e.g., FFPE) tissue and does not require any specific tissue processing or treatment. Therefore, it can be applied to archive biopsy tissue and could be easily translated into a clinical environment. Prospective validation and optimization of the QIF-based signature is ongoing and will require maturation of the cohort to evaluate the effect on long-term survival.

A rather unexpected finding was the lack of prominent benefit from immune checkpoint therapies in patients with tumors containing predominantly elevated T-cells with high effector markers and proliferation. One possible explanation for this is the limited potential for additional activation of this TIL population and the existence of alternative local immune suppression pathways in tumors with effective antigen recognition, activation and T-cell migration. In this regard, diverse mechanisms have been proposed to be used by tumors to block the T-cell attack including collagen deposition around tumor cells^40^, exacerbated lysosome secretion and perforin degradation at the lytic synapse^41^, cytotoxic T-cell trapping in specific tissue compartments^42^ and metabolic alterations of T-cells^43^.

To further support the role of PD-1 blockade in stimulating “dormant” TILs, we could experimentally increase the cytolytic activity and proliferation of extracted lymphocytes after treatment with a PD-1 blocking antibody in a patient-derived xenograft model. Interestingly, T-cell proliferation was more associated with a CD4+/T-BET+ cell phenotype and production of GZB was seen more frequently in CD8+/EOMES+ cells. A population of CD3+ cells showed both proliferation and cytolytic activity upon PD-1 blockade and was characterized by predominant CD8 positivity with co-expression of T-BET, EOMES, TIM-3, and HLA-DR. The latter indicate that different T-cell subsets show differential response to PD-1 antibodies. A similar result showing increased proliferation (e.g., “proliferative burst”) of antigen specific CD8+/CXCR5+ cells with a distinct transcriptional profile and lower levels of effector molecules was found in a murine model of chronic LCMV infection^44^. Future studies performing in-depth analyses of specific T-cell subpopulations using different NSCLC variants and spanning various treatment courses will be required to understand the dynamics of the adaptive response induced by PD-1 blockade.

A limitation of our study is the use of a cohort of NSCLC patients treated with different immune checkpoint blockers. Although all cases received PD-1 axis inhibitors, seven cases were additionally treated with concurrent CTLA-4 blockade. To date, most available data indicate that treatment with PD-1 axis inhibitors targeting PD-1 or PD-L1 produce comparable effects in NSCLC^1–4^. Other limitations of our study include heterogeneity of tumor sites analyzed and variable time points of tumor collection. Indeed, 59% of tumor specimens in the cohort were collected prior to other intervening anti-cancer systemic therapies and the median time from collection to initiation of immunotherapy was 9 months. Although the optimal time window or tumor site to reliably measure predictive immune-related biomarkers in lung cancer specimens has not been determined, our results suggest that key biological information is present in archived FFPE tumor specimens from varying sites not immediately preceding immune checkpoint blockade. Future studies analyzing prospectively collected samples and large cohorts will be required to clarify possible differences and their implications.

In summary, we have identified a TIL signature characterized by “dormant” or inactive TILs that is strongly associated with clinical benefit to immune checkpoint blockers in NSCLC patients and is independent from the tumor mutational load and PD-L1 expression. Additional studies will be required to determine the value of this signature as a clinical biomarker.

## Methods

### Cases and samples

We studied 49 pre-treatment FFPE samples from NSCLC patients who initiated immune checkpoint blockers between 2009 and 2014 at our institution. Cases were obtained from Yale Pathology archive and clinico-pathological information was extracted from the clinical records. Detailed description of the cohort is shown in the Supplementary Table 2. Twenty-nine cases were treated with PD-1 antibody monotherapy (nivolumab/pembrolizumab), 12 cases with PD-L1 antibody (atezolizumab), seven cases with dual PD-1/CTLA-4 blockade (nivolumab/ipilimumab or durvalumab/tremelimumab) and one case with concurrent nivolumab and erlotinib after progression on erlotinib alone. For the analyses and as previously reported^13^, cases showing partial or complete response by RECIST v1.1 lasting >24 weeks or stable disease lasting >24 weeks were considered as having durable clinical benefit (DCB). Cases showing progression of disease or stable disease lasting equal or less than 24 weeks were considered as showing no durable benefit (NDB).

A retrospective collection containing 110 NSCLCs not treated with immunotherapy and represented in TMA format was also included. Cases in TMAs were evaluated in 2-fold redundancy and using two independent blocks including cores from different tumor areas. Therefore, the results presented included integrated data from 2 to 4 independent tumor cores stained at least twice. Detailed description of this cohort is provided in the Supplementary Table 3. TMAs were prepared using 0.6 mm cores, each in 2-fold redundancy using standard procedures. Informed consent was obtained from the subjects and samples were used after approval from the Yale Human Investigation Committee protocols #9505008219, #0304025173, #1603017333, and #1412015109.

### Whole exome sequencing and mutational analysis

Genomic DNA from tumor and normal samples was captured on the Nimblegen 2.1M human exome array and DNA libraries were sequenced on the Illumina HiSeq2500 instrument using 74-bp paired-end reads. Sequence reads were mapped to the human b37 reference genome using the Burrow-Wheeler Aligner-MEM (BWA-MEM)^45^ program and mutation calling was performed with GATK following the Best Practices guidelines. For matched tumor-normal pairs, somatic point mutations and indels were called by MuTect2 using Bayesian classifiers. For unmatched tumor samples, MuTect2 compared the tumor to the reference panel of normal samples of the same ethnicity. For all somatic mutations called, the mutations that were supported by at least two alternative non-reference alleles present in more than 5% of all sequencing reads or a total of eight independent reads were considered. Identified variants were further filtered based on their presence in repositories of common variations (1000 Genomes, NHLBI exome variant server and 2577 non-cancer exomes sequenced at Yale) and annotated using ANNOVAR^46^. For all matched samples, somatic CNVs were analyzed using EXCAVATOR^47^ that normalizes the non-uniform whole-exome sequencing read depths taking GC-content, mappability, and exon-size into account and calculates the ratio of normalized read depth between tumor and normal for the exome capture intervals. LOH calling and purity estimation were performed as previously described^48^.

### HLA typing and class-I/II neoantigen prediction

The 4-digit patient-specific HLA class I type was determined by ATHLATES in silico^49^. All nonsynonymous somatic mutations identified from the whole exome sequencing analysis were translated into 17-mer polypeptides flanking the mutant amino acid. The binding affinity of mutant nonamers to the patient-specific HLA class I type was predicted using NetMHCcons algorithms, as previously reported^13,16,17,23^. Nonamers with IC50 below or equal to 500 nM were further tested for the recognition by the T-cell receptor using Class I immunogenicity^25^ resulting in putative neoantigens. Candidate MHC class-I neoantigens with calculated IC_50_ ≦ 50 nM were considered as high affinity binders, those with calculated IC_50_ between 50 ≦ 150 nM) were considered as intermediate binders and sequences with calculated IC_50_ > 150 ≦ 500 nM were considered as weak buinders. HLA class II type for each patient was estimated using the PHLAT algorithm. 53-mer polypeptides were identified by the in-house script with the nonsynonymous somatic mutation in the middle at position 27. The binding affinity of 53-mer polypeptides to the patient-specific HLA class II type was calculated by NetMHCIIpan-3.0.

### In vitro HLA-A2 stabilization assay

Experimental validation of the HLA binding capacity of in silico predicted class-I mutant neoantigenic peptides identified in NSCLC was performed by measuring the stabilization of HLA-A2 protein after incubation of B-lymphoblastoid LCL-174 cells with recombinant mutant 9-mer peptides. LCL-174 cells (available in the laboratory of Dr. Paula Kavathas, Yale University) are irradiated and immuno-selected human cells having specific deletions and lacking MHC-II genes and TAP proteins; and expressing only HLA-A2, -C1, and -B5 protein^50^. Cells were incubated overnight with 50 μM recombinant 9-mer mutant peptides found in the tumor sample of patient YLR029 and stained for HLA-A2 protein using a fluorescently labeled primary antibody by flow cytometry. Peptides inducing surface HLA-A2 signal above the negative control sample were considered as positive binders. Cell lines used in this study were authenticated every 3–6 months using the GenePrint® 10 System in the Yale University DNA Analysis Facility. Cells were also periodically tested for mycoplasma contamination.

### In vitro expansion of neoantigen specific T-cells and flow cytometry

PBMC were isolated from the lung cancer patient YLR029. Fourteen putative neoantigens predicted to bind A*02:01 HLA-I allele with binding affinities from 10–414 nM were synthesized and divided into two pools of eight (peptide pool #1 [TMOD4, TENM2, MDM2, NXPE1, RHOT2(A), RHOT2(I), KIF5A, ARHGAP9]) or six peptides (peptide pool #2 [GCN1L1, TBXAS1, GRIN3A, HSF1, CSGALNACT2, DUSP10]). In vitro expansion of CD8 T cells specific to the two peptide pools was performed using a protocol adapted from Greenberg et al.^51^, with some modifications. Briefly, autologous monocytes (adherent cells) were isolated from PBMC using the CD14 MicroBeads (Miltenyi Biotec). Monocytes were differentiated into dendritic cells (DCs) by IL-4 (25 ng/ml) and GMCSF (100 ng/ml) treatment for 7 days. The DCs were further matured by stimulation with lipopolysaccharides (LPS, 10 ng/ml) for 16 h. During the last 4 h of LPS stimulation, DCs were pulsed with 5 nM of the peptide pools and were subsequently co-cultured with lymphocytes. IL-7 (25 ng/ml) was added on the first day of co-culture and IL-2/IL-15 (100 U/ml/25 ng/ml) was added on days 2 and 5. After seven days of this initial co-culture, cells (mainly non-adherent lymphocytes) were stimulated and co-cultured with an artificial APC (K562-HLA-A2+, 4–1BBL+) pulsed with appropriate peptides pools (5 nM for 3 h.), irradiated (45 Gy), and mixed at 1:2 effector cell: APC ratio. IL-7 was added on the first day and IL-2/IL-15 was added on the second and the fifth day. At the end of this seven-day period, flow cytometry/intracellular cytokine staining assay was performed as previously described^52^. Briefly, 5 × 10^5^ cells were stimulated with the K562 artificial APC (10^6^ cells) that was pulsed either with pool #1, pool #2, or no peptide. After 30 min, brefeldin A (BioLegend) was added to the culture and stimulation continued for another 5.5 h. Then, the cells were stained with a viability dye (Invitrogen), as well as with anti-CD3-Percp Cy5.5, anti-CD4-PE, and anti-CD8-APC-Cy7 (BioLegend). Afterwards, the cells were permeabilized and stained with anti-IFN-γ-Pacific Blue and anti-TNF-α-FITC (BioLegend). At least 90,000 total events were acquired on an LSRII flow cytometer (BD Immunocytometry Systems) and data were analyzed using FlowJo software (version 10.2; Tree Star Inc.).

### DNA and RNA sequencing analysis from TCGA dataset

We analyzed gene expression and somatic mutation for 514 lung adenocarcinomas and 504 lung squamous cell carcinomas from The Cancer Genome Atlas database (TCGA). Somatic mutational load was calculated as total number of mutations identified in each tumor sample. Normalized gene expression of tumor was downloaded from TCGA data portal and further analyzed to correct for batch effects using the MD Anderson GDAC’s MBatch website (http://bioinformatics.mdanderson.org/tcgabatcheffects). Spearman’s rank correlations were then calculated between the somatic mutational load and normalized expression levels of 9 immune-related genes using 464 LUAD and 178 LUSC samples that have both somatic mutation and gene expression data.

### Immunohistochemistry

PD-L1 IHC was stained using the FDA-approved PD-L1 IHC 22C3 pharmDx kit on the Dako Link 48 platform according to manufacturer recommendations using 4–5 μm thick whole tissue histology tumor preparations. The 22C3 antibody in this kit is provided already diluted at an unspecified ratio, was stained in the CLIA laboratory of the Department of Pathology at Yale University and scored by a trained pathologist using bright field microscopy and a semi-quantitative score. Values were expressed as percentage of tumor cells displaying predominant membrane signal.

### Multiplexed QIF

The multiplexed TIL staining protocol was performed using 5-color multiplex fluorescence with simultaneous detection of markers labeled using isotype specific antibodies as previously described by our group^53^. Briefly, histology sections from the cases were deparaffinized and subjected to antigen retrieval using EDTA buffer (Sigma-Aldrich) pH = 8.0 and boiled for 20 min at 97 °C in a pressure-boiling container (PT module, Lab Vision). Slides were then incubated with dual endogenous peroxidase block (DAKO #S2003) for 10 min at room temperature and subsequently with a blocking solution containing 0.3% bovine serum albumin in 0.05% Tween solution for 30 min. Slides were stained with 4′,6-Diamidino-2-Phenylindole (DAPI) for visualization of all cells, CK to detect tumor epithelial cells, CD3 for T-lymphocytes, GZB for T-cell cytolytic potential and Ki-67 as cell proliferation marker. Primary antibodies included CK clone AE1/AE3 (catalogue # M3515) from DAKO used with a concentration of 0.12 mg/ml, CD3 clone SP7 (catalogue # NB600-1441) from Novus biologicals dilution 1:100 (culture supernatant), GZB clone 4E6 (catalogue # ab139354) from Abcam with a concentration of 5 μg/ml and Ki-67 clone MIB1 (catalogue # M724029-2) from DAKO with a concentration of 0.46 μg/ml. Secondary antibodies and fluorescent reagents used were goat anti-rabbit Alexa546 (Invitrogen; 1:100 dilution), anti-rabbit Envision (K4009, DAKO, 1:100 stock dilution) with biotynilated tyramide/Streptavidine-Alexa750 conjugate (Perkin-Elmer); anti-mouse IgG1 antibody (eBioscience) with fluorescein-tyramide (Perkin-Elmer), anti-mouse IgG2a antibody (Abcam) with Cy5-tyramide (Perkin-Elmer). Residual horseradish peroxidase activity between incubations with secondary antibodies was eliminated by exposing the slides twice for 7 min to a solution containing benzoic hydrazide (0.136 mg) and hydrogen peroxide (50 µl).

### Tissue fluorescence measurement and scoring

Quantitative measurement of the fluorescent signal was performed using the AQUA® method that enables objective measurement of targets within user-defined tissue compartments^9,52^. Briefly, the QIF score of each target in CD3+ T-cell compartment was calculated by dividing the target pixel intensities by the area of CD3 positivity in the sample. Scores were normalized to the exposure time and bit depth at which the images were captured, allowing scores collected at different exposure times to be comparable. Stained slides were visually examined by a pathologist and defective samples or areas with staining artifacts were re-analyzed or excluded.

### Patient-derived xenograft model

Briefly, surgical specimens (10 mm^2^) from primary lung carcinomas were divided in 2 halves. A portion of each half was used for morphology studies and most of the tissue was implanted subcutaneously into the flank of NOD-scid IL2rgc−/− male mice. Mice were treated intraperitoneally with anti-hPD-1 mAbs (clone M3) or PBS at days 5 and 10. At day 12 mice were sacrificed and tumors were collected for analysis. All institutional and national guidelines for the care and use of laboratory animals were followed.

### Cell preparation and mass cytometry (CyTOF) analysis

Tumors were minced and mechanically dissociated with the GentleMACS Dissociator (Miltenyi Biotec) in the presence of RPMI1640 with 0.5% BSA and 5 mM EDTA. The resulting cell suspension was filtered using a 70-μm cell strainer (BD Falcon). Cells were centrifuged at 600 g for 7 min at 4 °C and re-suspended in PBS with 0.5% BSA and 0.02% NaN3. 2 × 10^6^ cells from each tumor were incubated with antibodies against CD16/32 at 50 μg/ml in a total volume of 50 μl for 10 min at RT to block Fc receptors. Surface marker antibodies were then added, yielding 100 μL final reaction volume and stained for 30 min at 4 °C. Following staining, cells were washed twice with PBS containing 0.5% BSA and 0.02% NaN_3_. Then, cells were re-suspended with RPMI1640 and 10 μM Cisplatin (Fluidigm Corp) in a total volume of 400 μl for 1 min before quenching 1:1 with pure FBS to determine viability. Cells were centrifuged at 600 g for 7 min at 4 °C and washed once with PBS with 0.5% BSA and 0.02% NaN3. Cells were then fixed using Fixation/Permeabilization Buffer (ebioscience) for 30 min at 4 °C. After two washes with Perm buffer (ebioscience) cells were incubated with intracellular antibodies cocktail in 100 μl for 30 min at 4 °C. A summary of the antibodies/clones used in the mass cytometry analyses is presented in the Supplementary Table 4. Cells were washed twice in PBS with 0.5% BSA and 0.02% NaN3 and then stained with 1 mL of 1:4000 191/193Ir DNA intercalator (Fluidigm) diluted in PBS with 1.6% PFA overnight. Cells were then washed once with PBS with 0.5% BSA and 0.02% NaN3 and then two times with double-deionized (dd)H_2_O. Mass cytometry samples were diluted in ddH_2_O containing bead standards to approximately 10^6^ cells per mL and then analyzed on a CyTOF 2 mass cytometer (Fluidigm). All files were normalized together using the mass-cytometry data normalization algorithm^54^ and analyzed using viSNE.

### Statistical analyses

Mutational data and QIF signals were analyzed using Spearman’s Rho rank regression functions. Case data and characteristics were compared using non-parametric t-test for continuous variables and chi-square or Fisher’s exact test for categorical variables. Overall survival functions were compared using Kaplan–Meier estimates and statistical significance was determined using the log-rank test. The mutational load, candidate class-I neoantigen content and TIL markers were stratified using the median score. Statistical analyses were performed using JMP® Pro software (version 11.0, SAS Institute Inc.) and GraphPad Prism v6.0 (GraphPad Sofware, Inc).

### Data availability

The DNA sequencing data have been deposited in the NIH database of Genotypes and Phenotypes (dbGaP) (https://www.ncbi.nlm.nih.gov/gap) under the accession code phs001618.v1.p1. The DNA and RNA sequencing data from The Cancer Genome Atlas referenced during the study are available in a public repository from the http://cancergenome.nih.gov/. website. The authors declare that all the other data supporting the findings of this study are available within the article and its supplementary information files and from the corresponding author upon reasonable request.

## Electronic supplementary material

Supplementary Information
