# Supplementary material for: A dormant TIL phenotype defines non-small cell lung carcinomas sensitive to immune checkpoint blockers
Source: Nat Commun. 2018 Aug 10;9:3196. doi: 10.1038/s41467-018-05032-8 (PMC6086912; doi:10.1038/s41467-018-05032-8)
Supplement: Supplementary file 1 — Supplementary Information [file 41467_2018_5032_MOESM1_ESM.pdf]

Title: A dormant TIL phenotype defines non-small cell lung carcinomas (NSCLCs) sensitive to immune checkpoint blockers.

Gettinger et al.

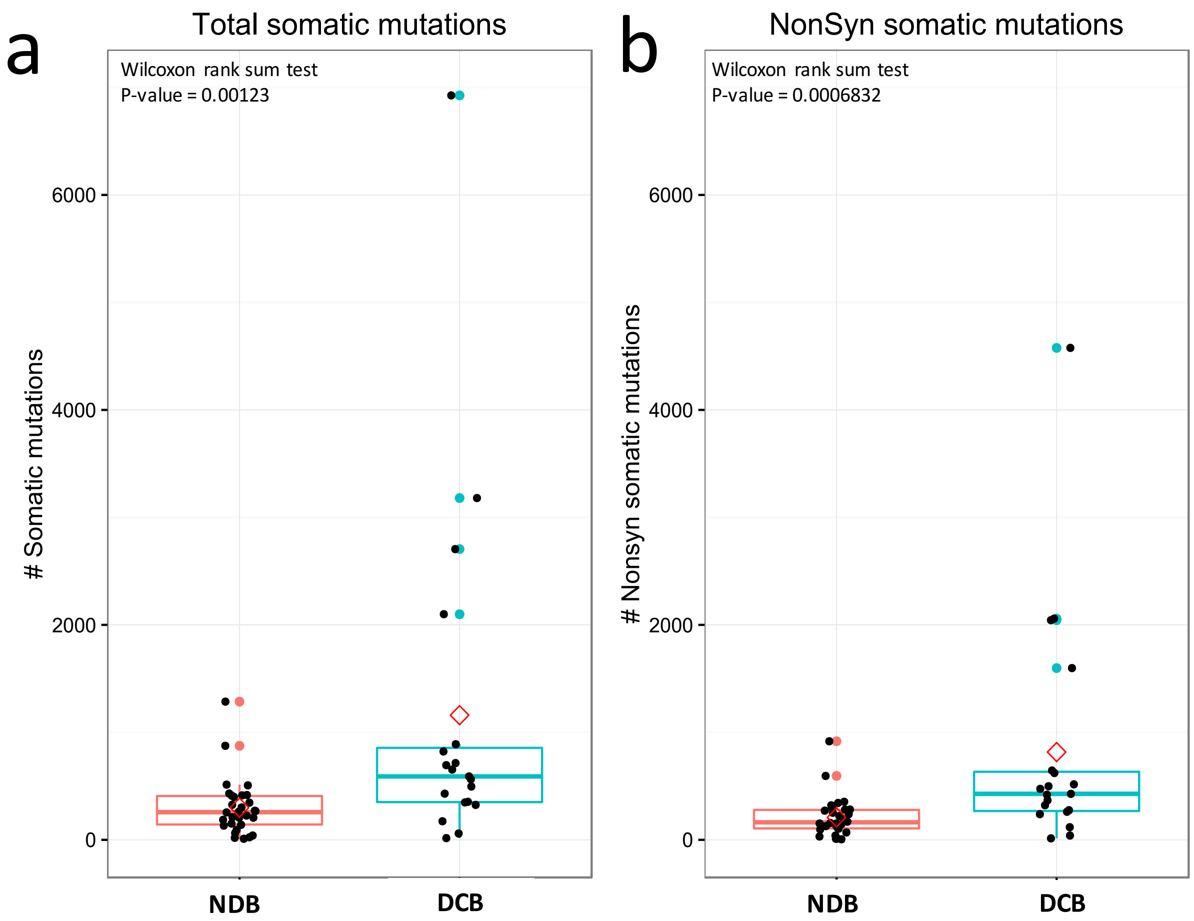

	Median	Mean
DCB	590	1160.0
NDB	257.5	299.7

	Median	Mean
DCB	428.0	816.8
NDB	163.0	209.0

Supplementary Figure 1. Association between total somatic mutations, nonsynonymous mutations and response to immune checkpoint blockers in NSCLC. a-b) Box plots showing the number of total mutations (a) or nonsynonymous mutations (b) stratified by cases with no durable benefit (NDB) and with durable clinical benefit (DCB) in the NSCLC cohort. Boxes indicate the 25-75% scores and diamonds indicate mean values. Colored dots indicate outlier values and black dots indicate all the scores in the cohort. Tables below each chart indicate the median and mean mutation numbers. P values and statistical for comparison of scores between groups are indicated within the chart.

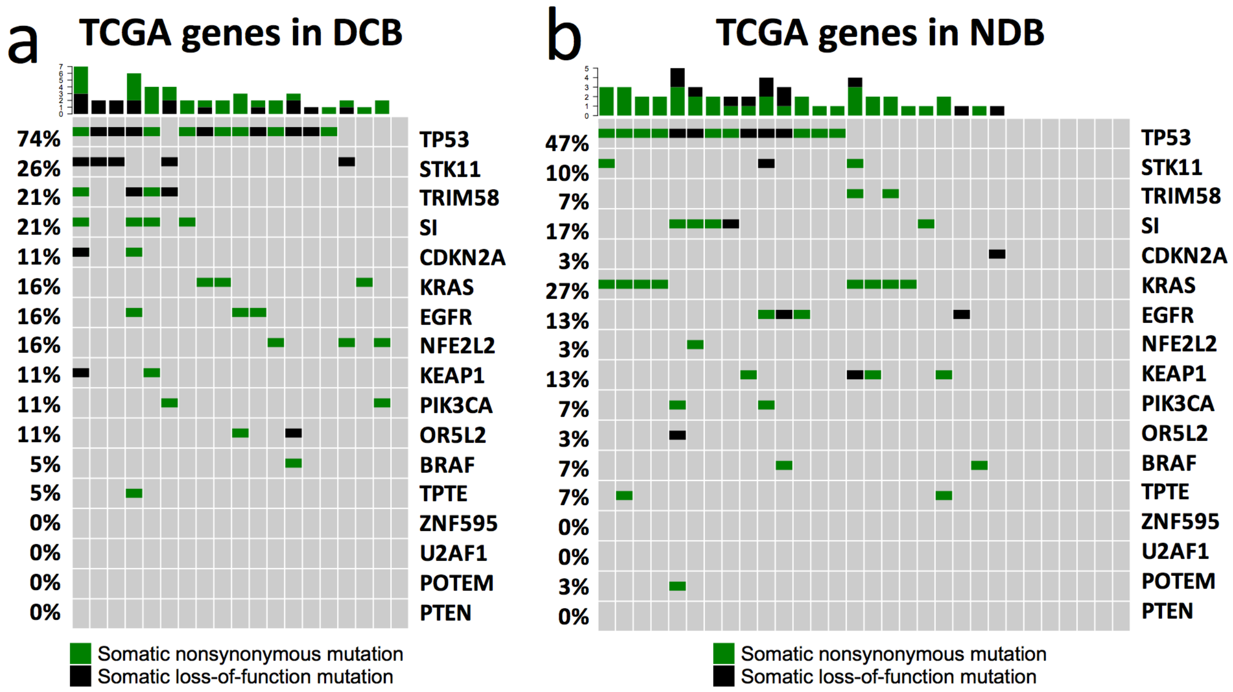

Supplementary Figure 2. Association between mutations in cancer-related genes from TCGA and sensitivity to immune checkpoint blockers in NSCLC. a-b) Graphical representation and frequency of nonsynonymous mutations in cancer-related genes of patients with NSCLC treated with immune checkpoint blockers with durable clinical benefor (**DCB, a**) or with no durable benefit after treatment (**NDB, b**). Green boxes indicate nonsynonymous mutations and black boxes indicate variants predicted to induce loss of function.

a

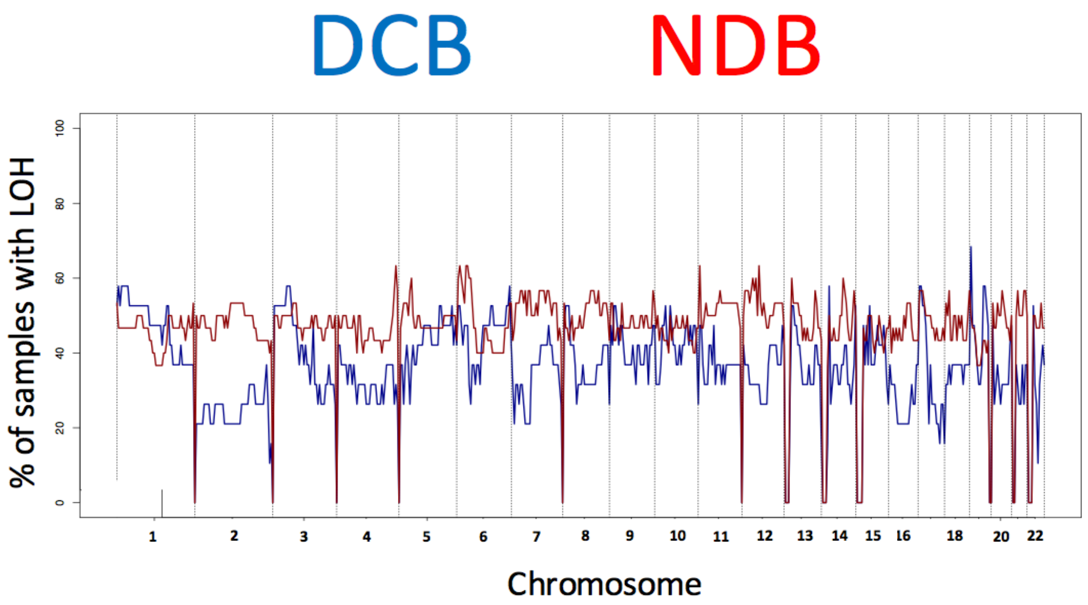

b

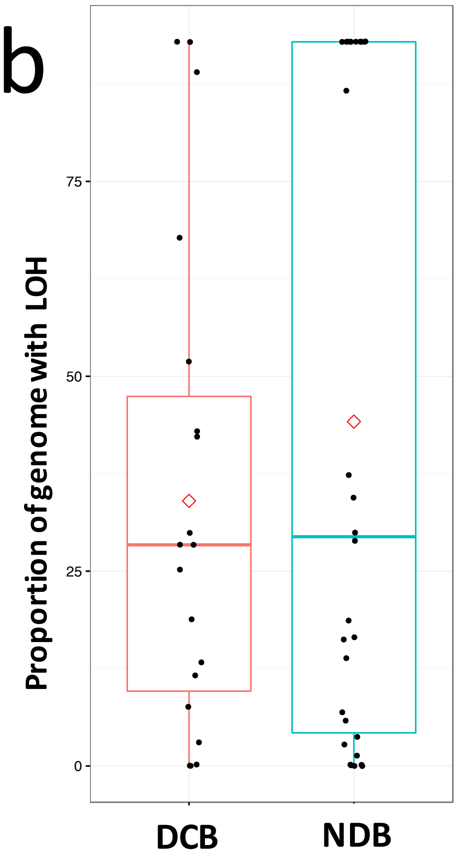

	Median	Mean
**DCB**	28.38	34.01
**NDB**	29.42	44.18

Supplementary Figure 3. Association between loss of heterozygosity (LOH) and response to immune checkpoint blockers in NSCLC. a) Graphical representation and frequency of LOH by chromosome in patients with NSCLC treated with immune checkpoint blockers with durable clinical benefor (DCB, blue line) or with no durable benefit after treatment (NDB, red line). b) Box plots showing the proportion of the genome with LOH in cases with durable clinical benefit (DCB) and no durable benefit (NDB) from the cohort. Boxes indicate the 25-75% scores and diamonds indicate mean values. Tables below each chart indicate the median and mean LOH proportion. Difference between the groups was not statistically significant (P=0.5216, Wilcoxon rank sum test). The error bars indicate the standard deviation.

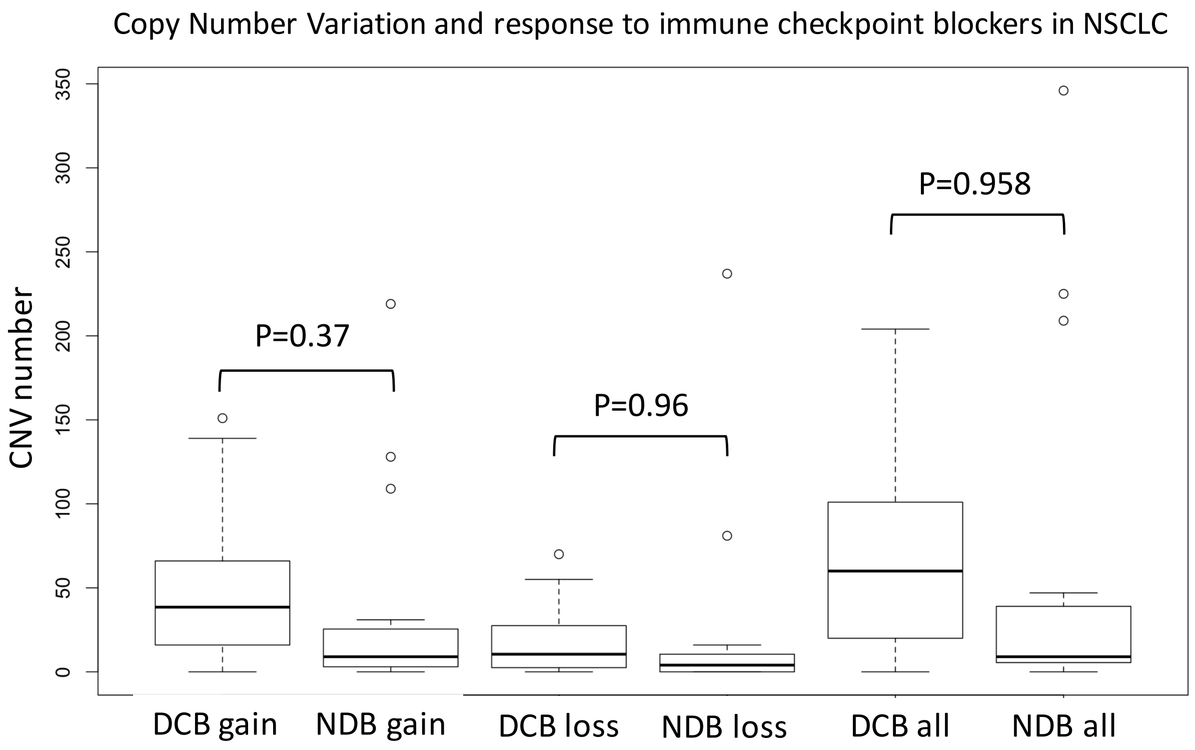

Supplementary Figure 4. Association between genetic copy number variation (CNV) and response to immune checkpoint blockers in NSCLC. Graphical representation of the number of loci with CNV across the exome in patients with NSCLC treated with immune checkpoint blockers with durable clinical benefor (DCB) or with no durable benefit after treatment (NDB). Cases were stratified based on copy number gains and losses. Difference between the groups was not statistically significant using the Wilcoxon rank sum test and P values are indicated within the chart. The error bars indicate the standard deviation.

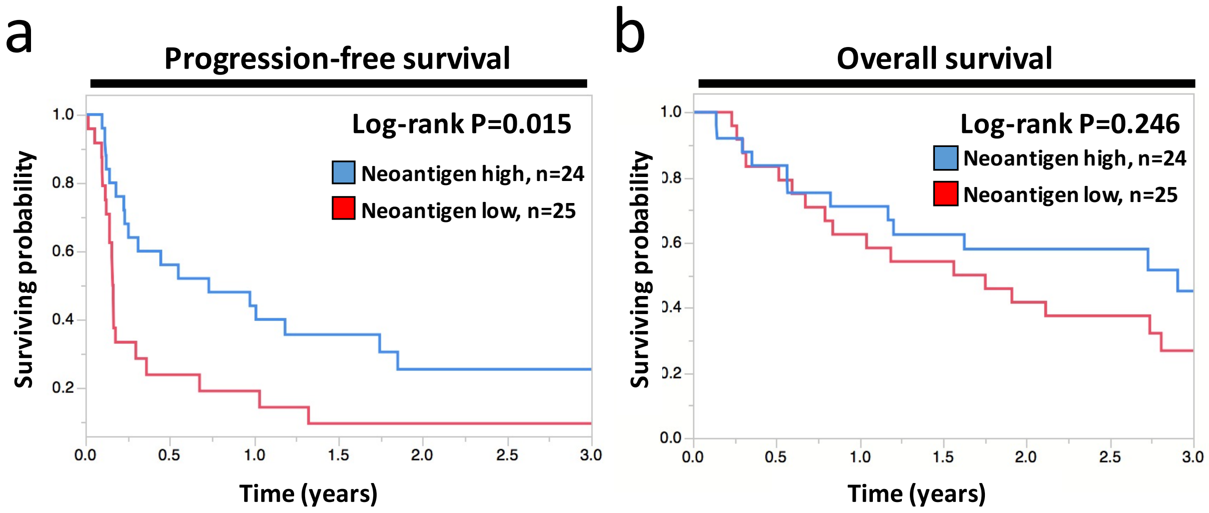

Supplementary Figure 5. Association between the level of candidate MHC-class I neoantigens and survival in NSCLC patients treated with immune checkpoint blockers. a-b) Kaplan-Meier graphical analysis of the 3-year progression-free survival (a) and overall survival (b) of NSCLC patients after treatment with immune checkpoint blockers. The median candidate MHC class-I neoantigen number was used as stratification cut point. The number of cases in eah group and log-rank P value are indicated within each chart.

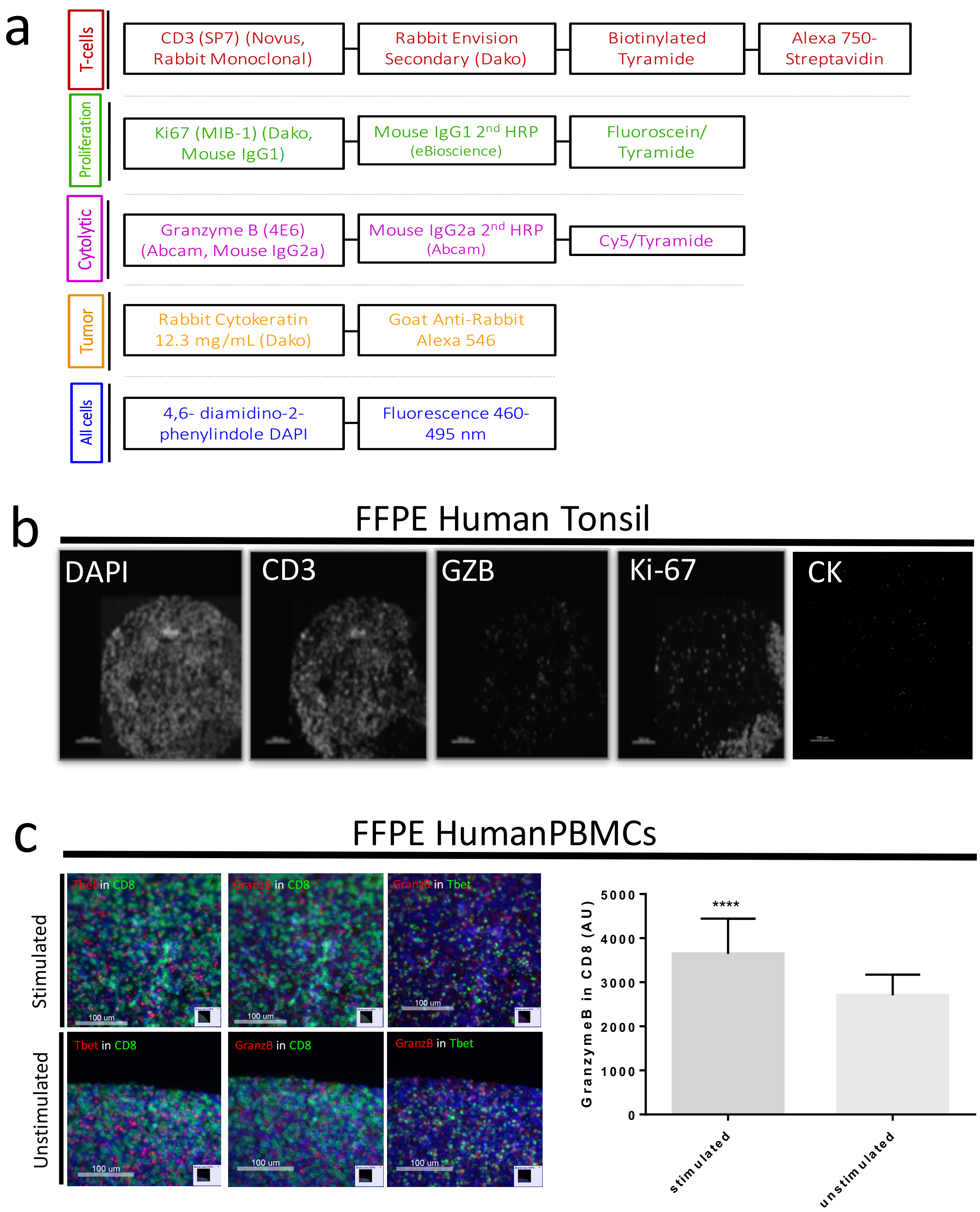

Supplementary Figure 6. Validation of a 5-color multiplex quantitative fluorescence assay to measure T-cell infiltration proliferation and cytolytic activity in NSCLC. a) Schema showing the primary, secondary and specific fluorophores used to stain the multiplexed panel

including markers for all cells in the sample (DAPI, blue), epithelial tumor cells (cytokeratin, yellow), T-lymphocytes (CD3, red), cell proliferation (Ki-67, green) and cytolytic granules (Granzyme-B, magenta). b) Localized expression of each of the markers in te panel in FFPE samples form human tonsil. c) Simultaneous staining of Tbet, CD8 and Granzyme-B in FFPE samples form unstimulated peripheral human PBMCs or PBMCs stimulated for 72h with anti CD3 (clone OKT3) and CD28 (clone CD28.2) antibodies. The chart on the right shows the level of Granzyme-B measured in each sample using quantitative immunofluorescence. The results shows the average of at least 10-20x fields of view in 3 individual experiments. The bars indicate the S.E.M. ****=P<0.0001 using the Mann-Whitney test.

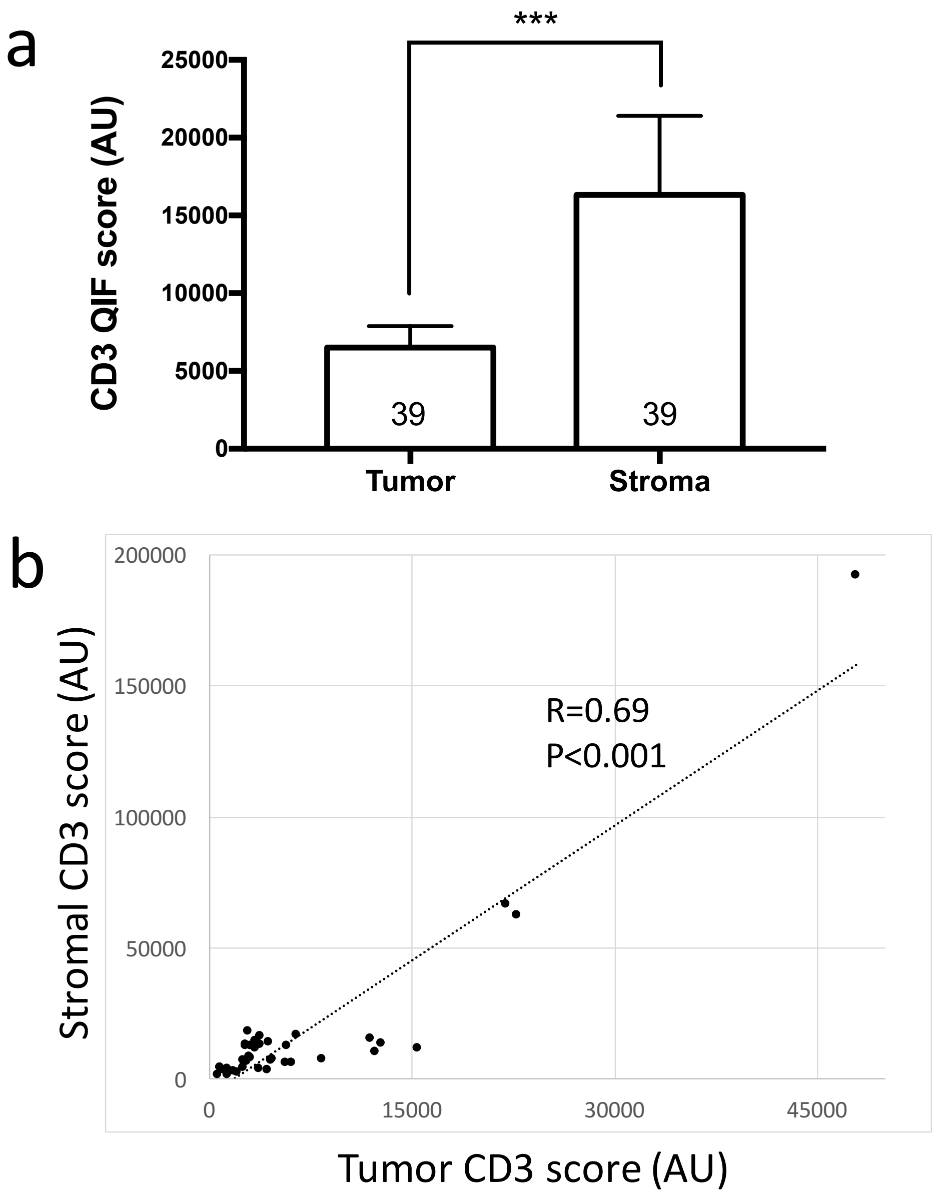

Supplementary Figure 7. Association between the level of CD3 in the tumor and stroma of NSCLC samples. a) Bar chart showing the level of CD3 selectively measured by QIF in the cytokeratin-positive tumor area and in the cytokerating-negative stromal compartment. The number of cases in each group is indicated within the bars. Error bars indicate the S.E.M. ***=P<0.001 uwing the Mann-Whitney test. b) Correlation between the level of CD3 in the cytokeratin-positive tumor area and cytokearatin-negative tumor compartment using QIF in FFPE preparations from NSCLC. R indicates the Spearman correlation coefficient and associated P value.

a

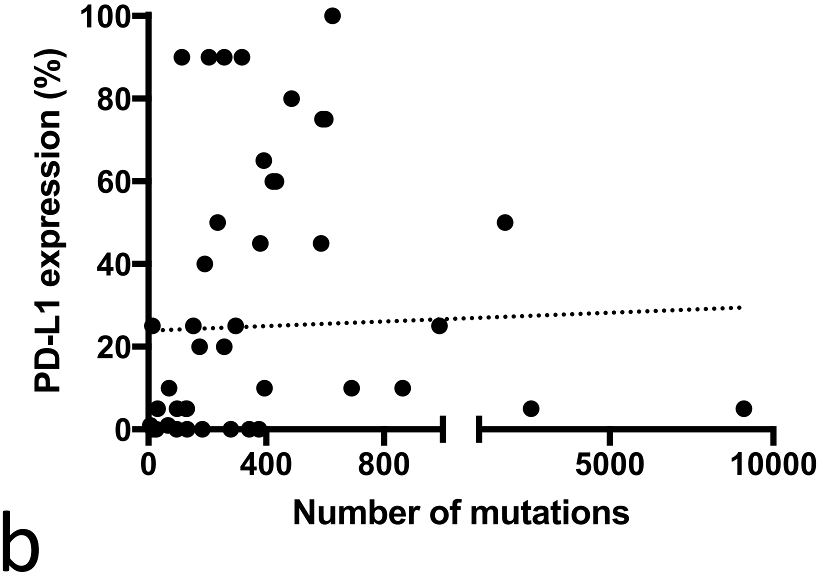

b

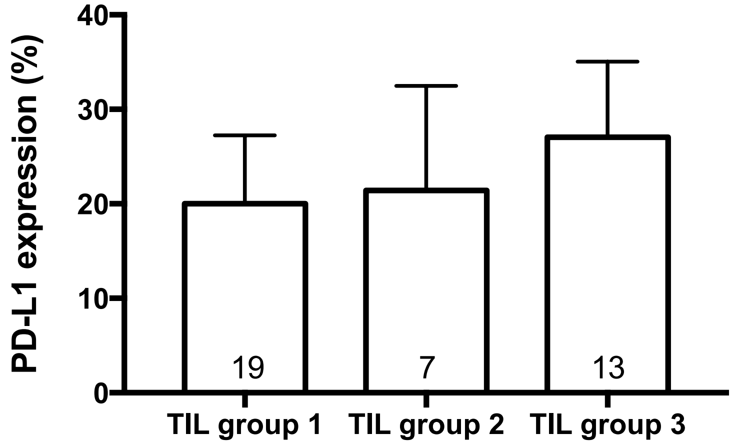

Supplementary Figure 8. Association between the mutational load, TILs subgroups and PD-L1 expression in NSCLC. a) Correlation between the number of nonsynonymous mutations and percentage of tumor PD-L1 expression in NSCLC cases from the cohort. b) Proportion of tumor cells positive for PD-L1 in NSCLC cases stratified by TIL groups. The number of cases in each group is indicated within the bars. Error bars indicate S.E.M. The difference between the groups was not statistically significant (P>0.05, one way ANOVA).

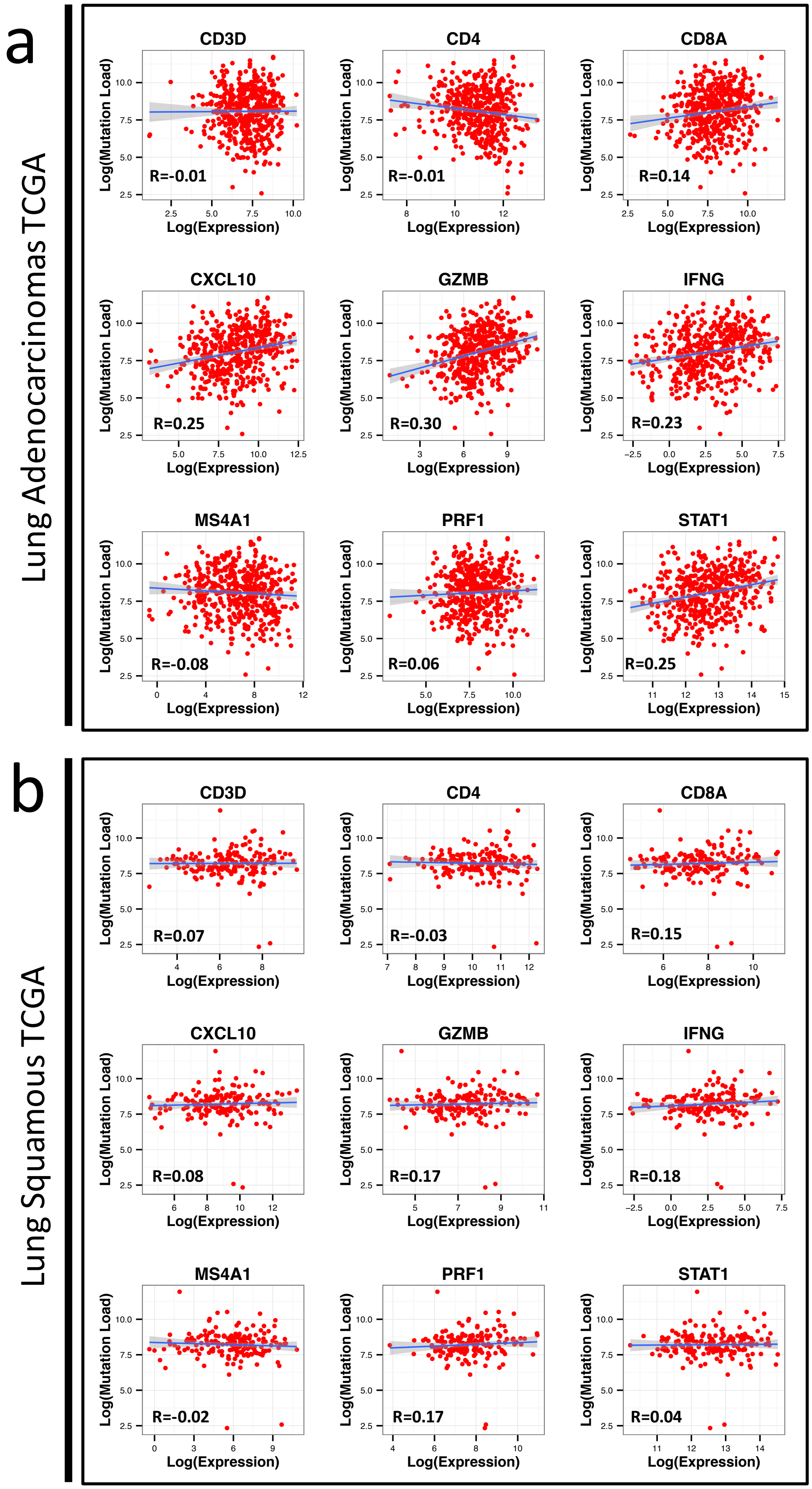
a
Lung Adenocarcinomas TCGA
CD3D
R=-0.01
CD4
R=-0.01
CD8A
R=0.14
CXCL10
R=0.25
GZMB
R=0.30
IFNG
R=0.23
MS4A1
R=-0.08
PRF1
R=0.06
STAT1
R=0.25
Log(Mutation Load)
Log(Expression)
b
Lung Squamous TCGA
CD3D
R=0.07
CD4
R=-0.03
CD8A
R=0.15
CXCL10
R=0.08
GZMB
R=0.17
IFNG
R=0.18
MS4A1
R=-0.02
PRF1
R=0.17
STAT1
R=0.04
Log(Mutation Load)
Log(Expression)

Supplementary Figure 9. Association between the mutational load and expression of diverse immune-related transcripts in NSCLC cases from TCGA. a-b) Charts showing the correlation between the number of somatic DNA mutations and the expression levels of diverse immune transcripts in lung adenocarcinomas (a) or in lung squamous cell carcinomas (b) from the TCGA cohort. The Spearman's correlation coefficient for each markers is indicated withing the charts.The number of mutations and transcript levels were log2 transformed.

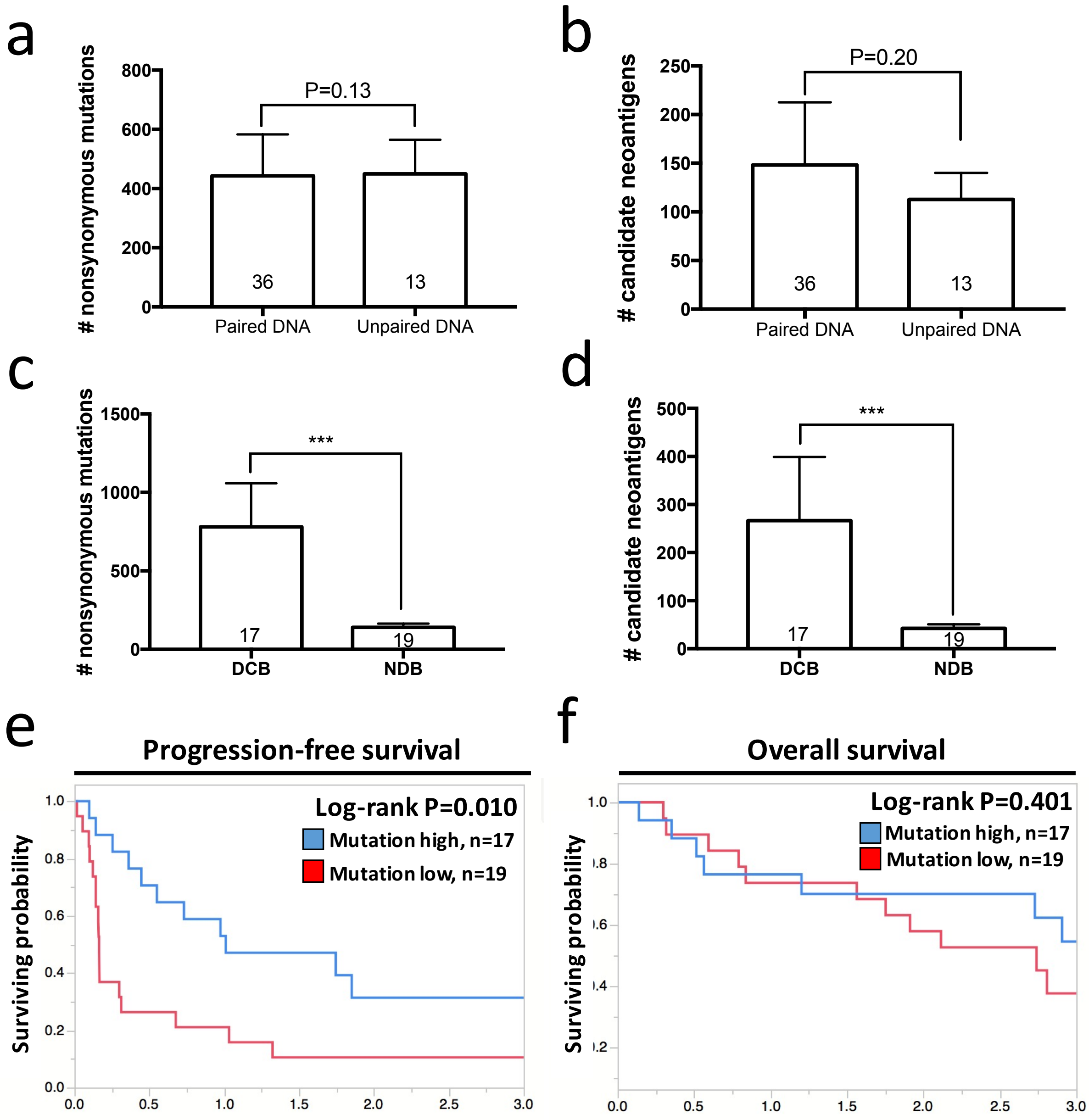

Supplementary Figure 10. Association between the mutational load, candidate MHC class-I neoantigens and outcome in NSCLC cases with paired germline/tumor DNA and excluding cases with unpaired DNA samples. a-b) Charts showing the number of nonsynonymous mutations (a) and candidate MHC class-I neoantigens (b) in cases with matched

germline DNA (paired DNA) and without available germline DNA (unpaired DNA). Error bars indicate S.E.M. The P value obtained using the Mann-Whitney test is indicated within the charts. c-d) Association between the mutational load (c) and predicted MHC class-I neoantigens (d) with durable clinical benefit (DCB) and no durable benefit (NDB) to immune checkpoint blockade in the cohort after excluding cases lacking paired germline DNA for the analysis. The number of cases in each group is indicated in each bar. Error bars indicate S.E.M. ***=P<0.001, Mann-Whitney test. (e-f) Association between the mutational load and 3-year progression-free survival (e) and overall survival (f) after treatment with immune checkpoint blockers excluding cases lacking paired germline DNA. The median mutational number was used as stratification cut point. The number of cases in each group and log-rank P value are indicated within each chart.

Supplementary Table 1. Level of strong, intermediate and weak candidate MHC class-I neoantigens in the NSCLC cohort of patients treated with immune checkpoint blockers.

	Neoantigen (n=49)			
	**Total**	**Strong (≦ 50 nM)**	**Intermediate (> 50 & ≦ 150 nM)**	**Weak (>150 & ≦ 500 nM)**
Median	72	16	20	34
Mean	138.78	35.94	37.14	65.69

Supplementary Table 2.. Clinico-pathologic characteristics of the cases in the NSCLC cohort treated with immune checkpoint blockers

Characteristic	Number (%)
**Age**	
≥70	16 (33)
<70	33 (67)
Median	65 years
**Gender**	
Male	25 (51)
Female	24 (49)
**Race/Ethnicity**	
White	44 (90)
Black	1 (2)
Asian	2 (4)
Hispanic	2 (4)
**Smoking History**	
Never Smoker	6 (12)
< 1 Pack Year	3 (6)
1 to 5 Pack Year	3 (6)
>5 to 10 Pack Year	1 (2)
>10 to 15 Pack Year	3 (6)
>15 to 20 Pack Year	7 (14)
>10 Pack Year	26 (53)
**Tumor Histology**	
Adenocarcinoma	33 (67)
Squamous carcinoma	10 (20)
Adenosquamous	1 (2)
Poorly Differentiated	5 (10)
**Oncogenic mutation**	
EGFR mutant	8 (16)
KRAS mutant	11 (22)
**Stage at Diagnosis**	
I	4 (8)
II	9 (18)
III	13 (27)
IV	23 (47)
**Tumor Sample Site**	
Primary	19 (39)
Metastatic, Lymph Node	14 (28)
Metastatic, Non- Lymph Node	16 (33)
**Sample Type**	
Biopsy	21 (43)
Resection	28 (57)
**Time from Tumor Collection to ICB treatment**	
≤ 4 weeks	8 (16)
> 4 weeks to 8 weeks	6 (12)
> 8 weeks to 24 weeks	5 (10)
> 24 weeks to 1 year	8 (16)
> 1 year to 2 years	12 (25)
> 2 to 3 years	6 (12)
> 3 years	4 (8)
Median	9 months
**Systemic therapy Between Tumor Collection and ICB treatment**	
Yes	29 (59)
No	20 (41)
**ICB Treatment**	
PD-1 blockade	29 (59)
PD-L1 blockade	12 (25)
PD-1 and CTLA-4 blockade	7 (14)
PD-1 and Erlotinib*	1 (2)
**Prior Systemic Therapy**	
None	7 (14)
1 Line	17 (35)
2 Lines	6 (12)
3+ Lines	19 (39)
**Prior Radiation**	
Yes	33 (67)
No	16 (33)

* After progression on erlotinib as last line of therapy

ICB=Immune checkpoint blocker

Supplementary Table 3. Clinico-pathologic characteristics of the cases in the NSCLC cohort not treated with immune checkpoint blockers.

Characteristic	Number (%)
**Age (Years)**	
< 70	59 (54.6%)
≥ 70	49 (45.4%)
**Gender**	
Male	55 (50.9%)
Female	53 (49.1%)
**Smoking Status**	
Never	6 (5.6%)
Smoker	102 (94.4%)
Unknown	0 (0.0%)
**Histology**	
Adenocarcinoma	62 (57.4%)
Squamous	23 (21.3%)
Other	23 (21.3%)
**Stage**	
0	8 (7.4%)
I-II	71 (65.7%)
III-IV	29 (26.9%)
Unknown	0 (0.0%)

Supplementary Table 4. Targets, antibodies and metal labels used in the mass cytometry analysis.

Chanel	Metal	Target	Clone	Vendor	#catalog	Volume**
141	Pr	HLA-ABC	W6-32	FDM	3141010B	1
142	Nd	CD19	HIB19	FDM	3142001B	0.5
143	Nd	CD127	A019D5	FDM	3143012B	1
144	Nd	CD69	FN50	FDM	3144018B	1
145	Nd	CD28	CD28.2	Biolegend*	302902	1.5
146	Nd	CD8a	RPA-T8	FDM	3146001B	0.3
147	Sm	CD11c	Bu15	FDM	3147008B	1
148	Nd	CD16	3G8	FDM	3148004B	1
149	Sm	CD45RO	UCHL1	FDM	3149001B	1.5
150	Nd	LAG3	11C3C65	FDM	3150030B	1
151	Eu	CD14	M5E2	FDM	3151009B	1.5
152	Sm	TCRgd	11F2	FDM	3152008B	2
153	Eu	CCR4	L291H4	FDM	3153030A	0.3
154	Sm	CD45RA	HI100	Biolegend*	304143	1
156	Gd	PD-L1	29E.2A3	FDM	3156026B	1
158	Gd	CD137	BBK2	Thermo Sci*	MA5-13739	0.1
159	Tb	CCR7	G043H7	FDM	3159003A	1.5
160	Gd	Tbet	4B10	FDM	3160010B	0.5
161	Dy	BCLXL	7B2.5	EM Millipore*	MAB3121	1
162	Dy	Foxp3	PCH101	FDM	3162024A	1
163	Dy	CXCR3	G025H7	FDM	3163004B	1
164	Dy	FAS (CD95)	DX2	FDM	3164008B	1
165	Ho	EOMES	WD1928	Thermo Fisher*	14-4877-80	1
166	Er	TIM-3	D5D5R	Cell Sig Tech*	45208S	1
167	Er	Gata3	TWAJ	FDM	3167007A	0.1
168	Er	Ki-67	Ki-67	FDM	3168001B	0.5
169	Tm	CD25 (IL-2R)	2A3	FDM	3169003B	0.5
170	Er	CD3	UCHT1	FDM	3170001B	1.5
171	Yb	Granzyme B	GB11	FDM	3171002B	0.3
172	Yb	mCD11b	M1/70	FDM	3172012B	0.5
173	Yb	HLA-DR	L243	FDM	3173005B	1
174	Yb	CD4	SK3	FDM	3174004B	0.2
175	Lu	PD-1	EH12.2H7	FDM	3175008B	1
176	Yb	CD56	NCAM16.2	FDM	3176008B	0.3
209	Bi	CD11b	ICRF44	FDM	3209003B	0.5
89	Y	CD45	HI30	FDM	3089003B	2

Supplementary Table 5. Multivariable Cox model of the survival effect of mutational burden, PD-L1 expression and TIL groups.

Variable	Risk Ratio [CI]	P value
Mutation number	0.89 [0.899-0.999]	**0.046**
PD-L1 IHC	0.91[0.986-1.016]	0.712
QIF group	0.581 [0.326-0.981]	**0.039**

The mutational load/number was stratified into high/low using the median score of the cohort. PD-L1 IHC scores were stratified using 1% tumor proportion score (TPS). The QIF groups were stratified into 3 groups as indicated in the results and methods section. Additional variables such as smoking status and level of CD3+ TILs were not included in the model due to their significant correlation with the mutation load and QIF groups, respectively.
